# Supplementary figures and images for: EPAC1 inhibition protects the heart from doxorubicin-induced toxicity
Source: eLife. 2023 Aug 8;12:e83831. doi: 10.7554/eLife.83831 (PMC10484526; doi:10.7554/eLife.83831)

**Fig. 1f**

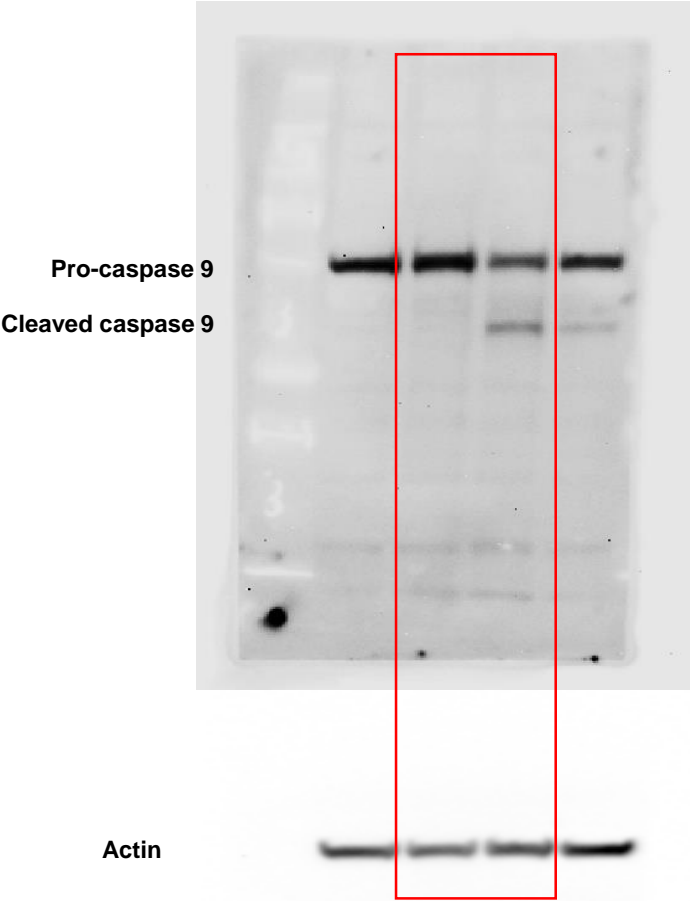

**Fig. 1g**

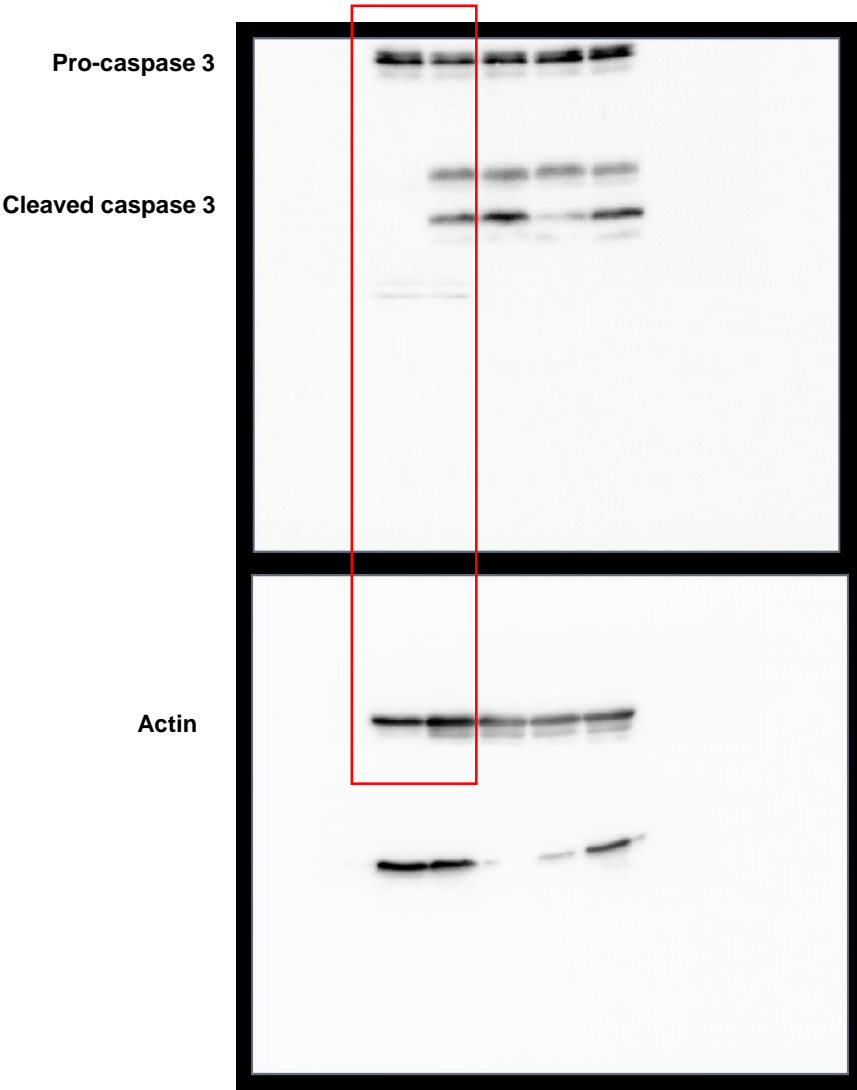

**Fig. 1j**

H<sub>2</sub>AX-pS139

Actin

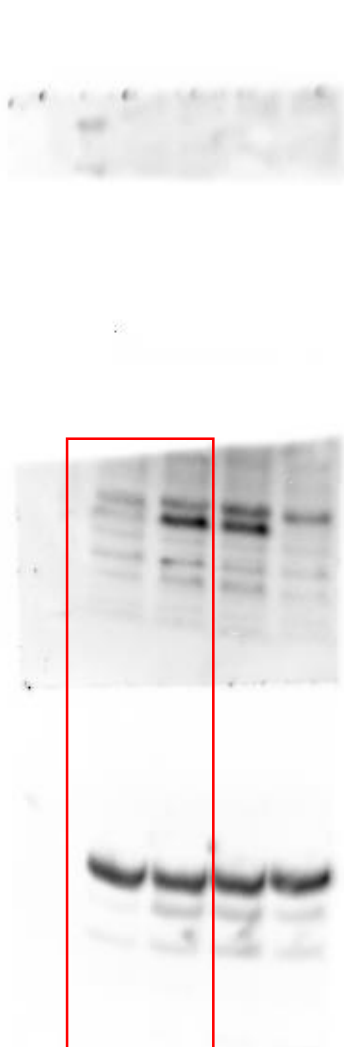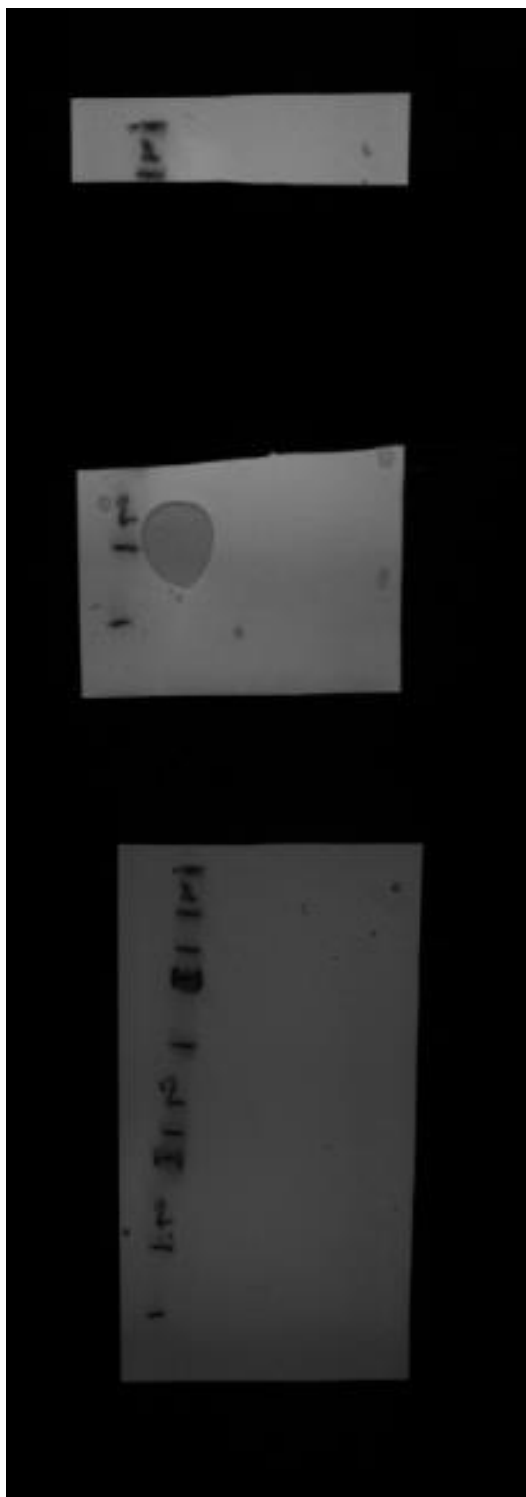

Supplement: Figure 1—source data 2. [file elife-83831-fig1-data2.zip › Data_WB_Fig1/Data_WB_Fig.1.pdf]

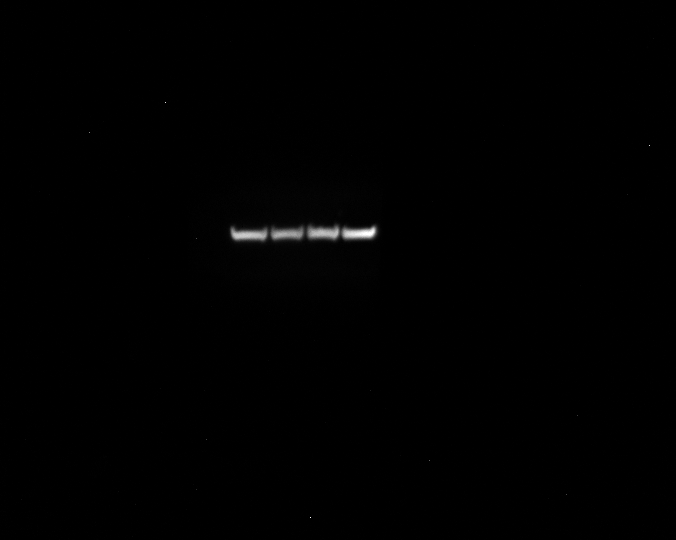

Supplement: Figure 1—source data 3. [file elife-83831-fig1-data3.zip › Figure 1_WB/Fig1f_WB/Actin-m3-2018-03-23_(Chemi)_raw.tif]

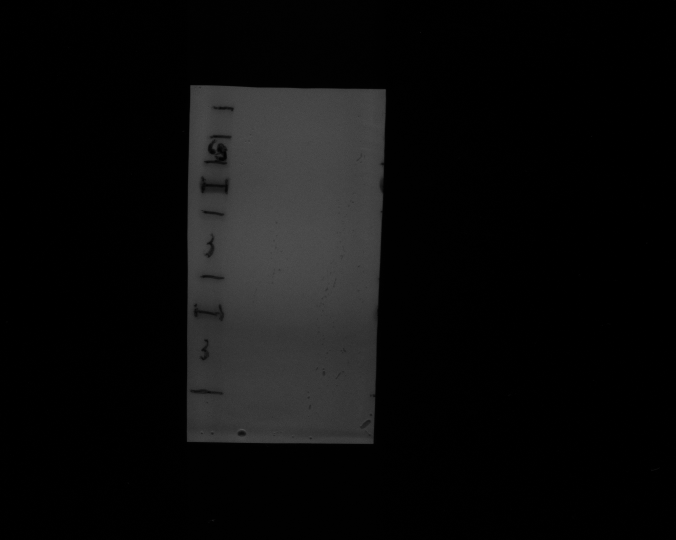

Supplement: Figure 1—source data 3. [file elife-83831-fig1-data3.zip › Figure 1_WB/Fig1f_WB/Actin-m3-2018-03-23_(Membrane)_raw.tif]

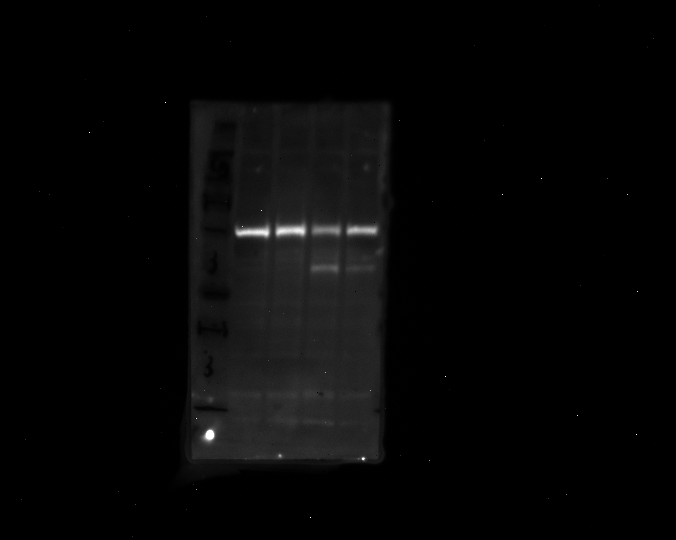

Supplement: Figure 1—source data 3. [file elife-83831-fig1-data3.zip › Figure 1_WB/Fig1f_WB/Caspase9-m3-2018-03-23_(Chemi)_raw.tif]

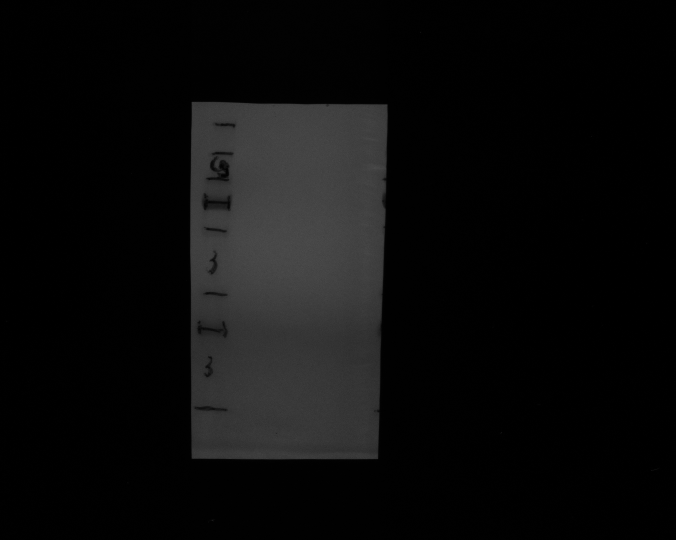

Supplement: Figure 1—source data 3. [file elife-83831-fig1-data3.zip › Figure 1_WB/Fig1f_WB/Caspase9-m3-2018-03-23_(Membrane)_raw.tif]

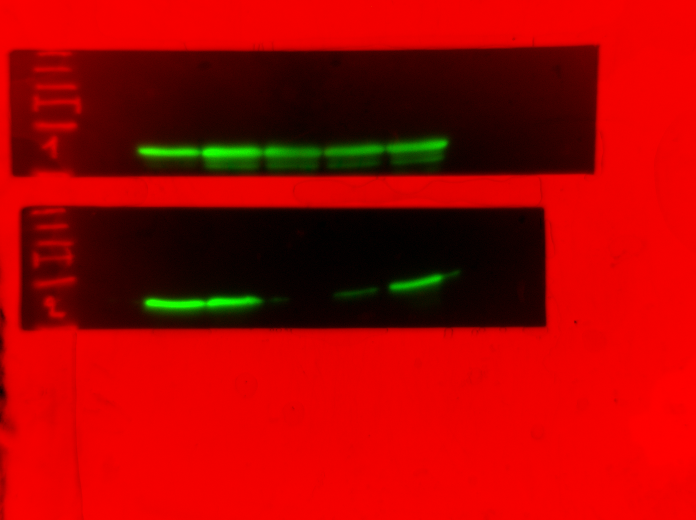

Supplement: Figure 1—source data 3. [file elife-83831-fig1-data3.zip › Figure 1_WB/Fig1g_WB/actin_Fig1g.tif]

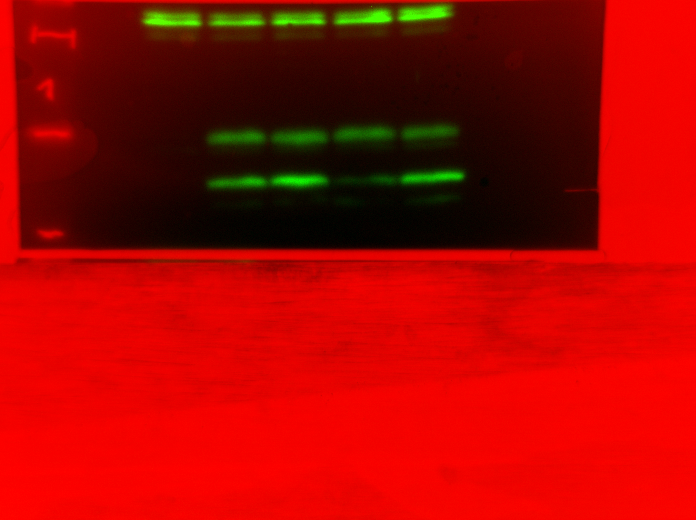

Supplement: Figure 1—source data 3. [file elife-83831-fig1-data3.zip › Figure 1_WB/Fig1g_WB/Caspase 3_Fig1g.tif]

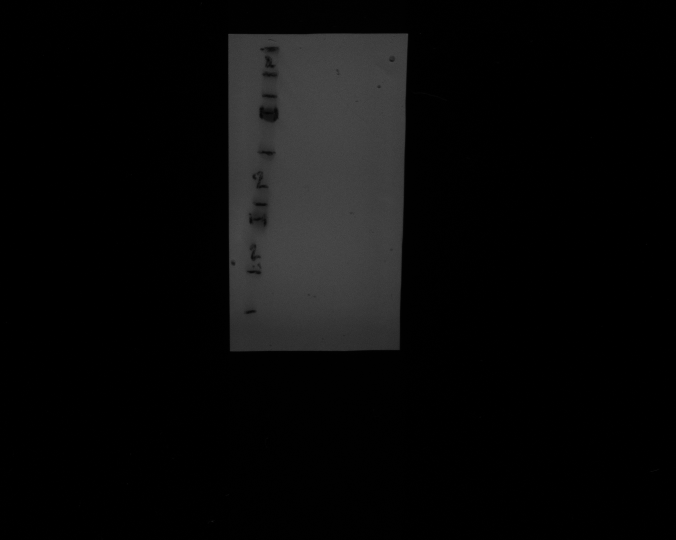

Supplement: Figure 1—source data 3. [file elife-83831-fig1-data3.zip › Figure 1_WB/Fig1j_WB/actin_Fig1j(Membrane).tif]

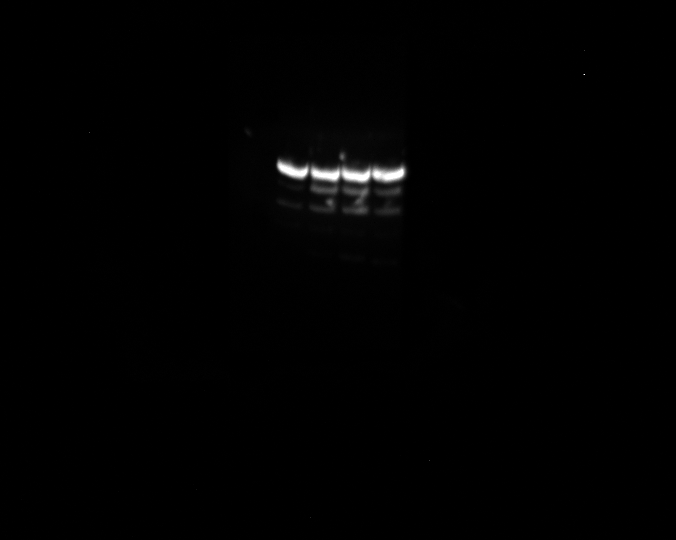

Supplement: Figure 1—source data 3. [file elife-83831-fig1-data3.zip › Figure 1_WB/Fig1j_WB/actin_Fig1j.tif]

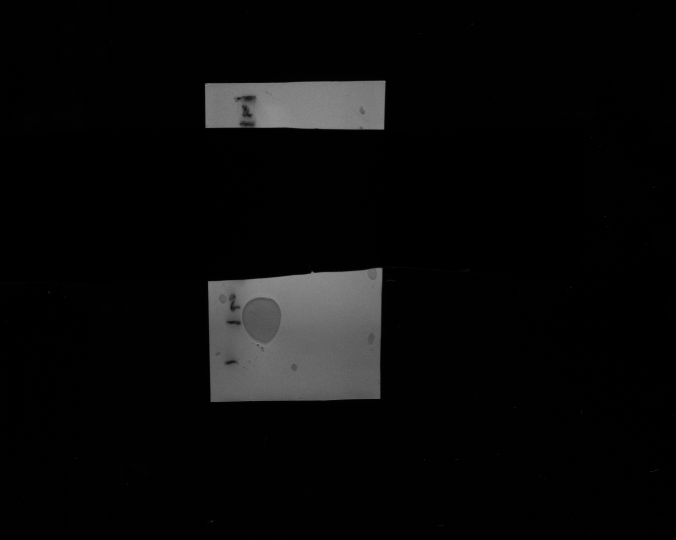

Supplement: Figure 1—source data 3. [file elife-83831-fig1-data3.zip › Figure 1_WB/Fig1j_WB/pH2AX_Fig1j(Membrane).tif]

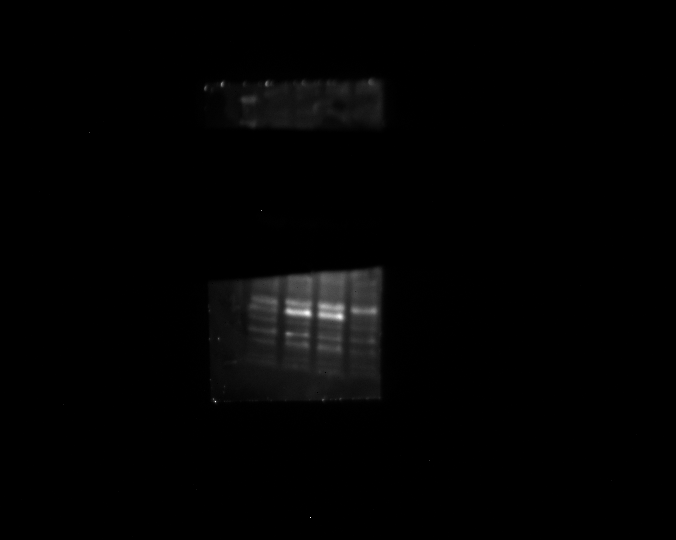

Supplement: Figure 1—source data 3. [file elife-83831-fig1-data3.zip › Figure 1_WB/Fig1j_WB/pH2AX_Fig1j.tif]

Fig. 2a

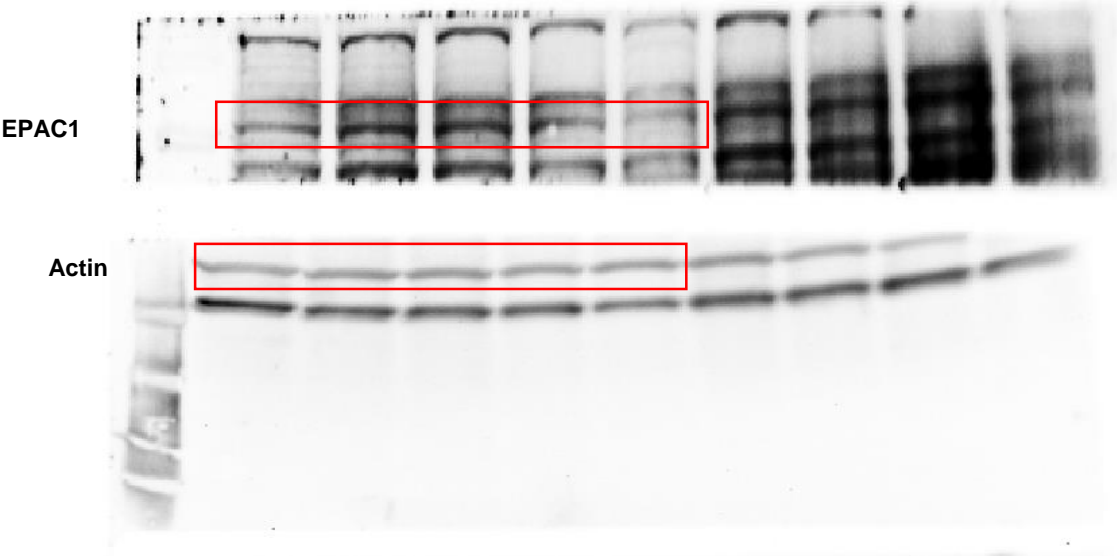

Fig. 2b

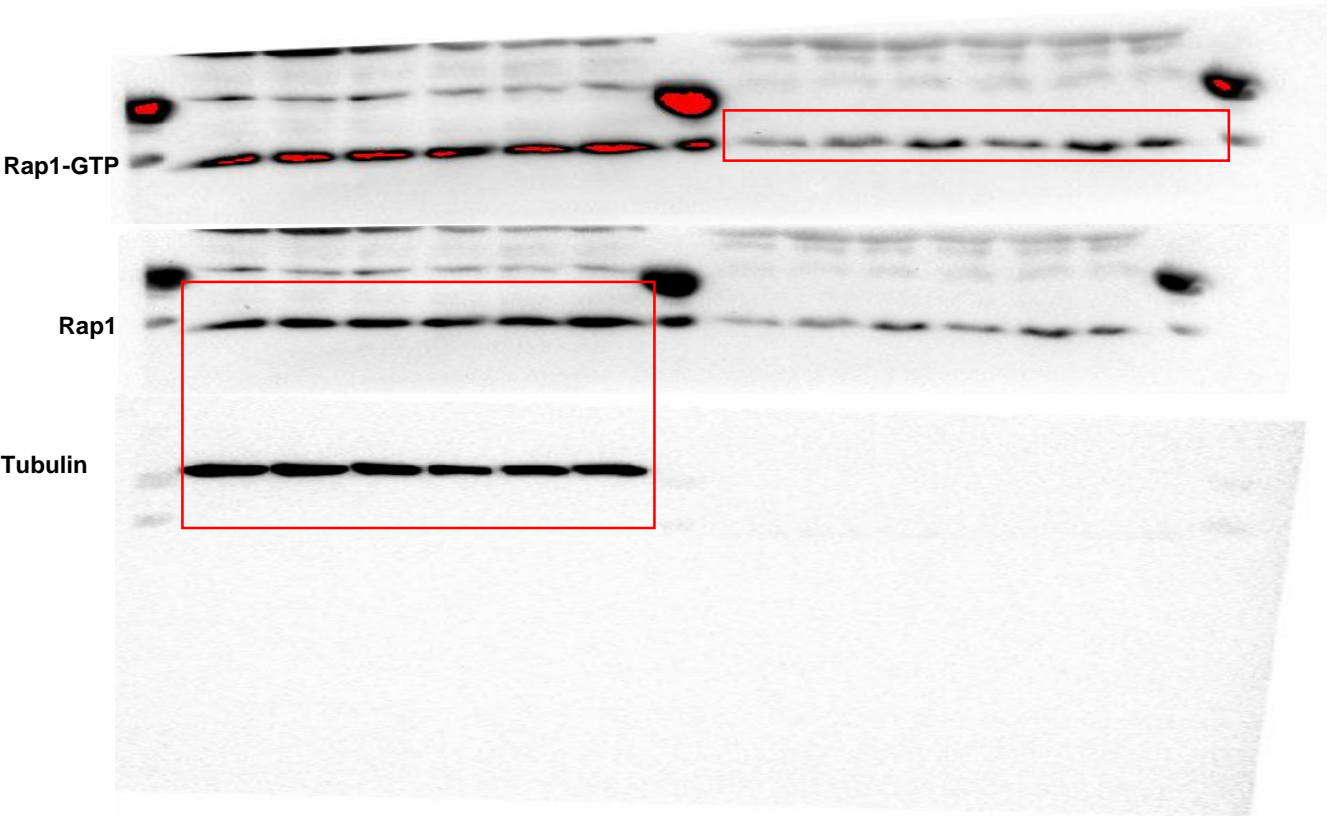

Supplement: Figure 2—source data 2. [file elife-83831-fig2-data2.zip › Data_WB_Fig2/Data_WB_Fig.2.pdf]

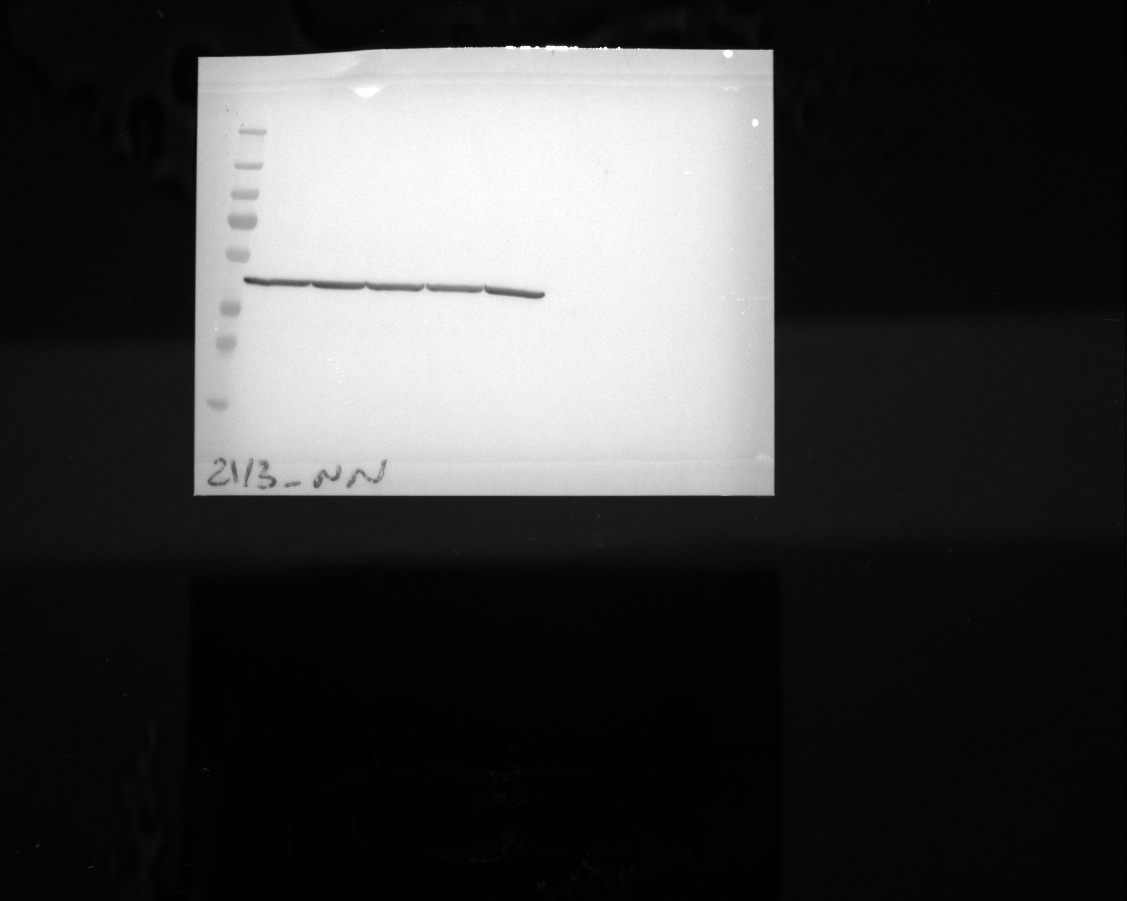

Supplement: Figure 2—source data 3. [file elife-83831-fig2-data3.zip › Figure 2_WB/Fig2a_WB/Actin_Fig2a.jpg]

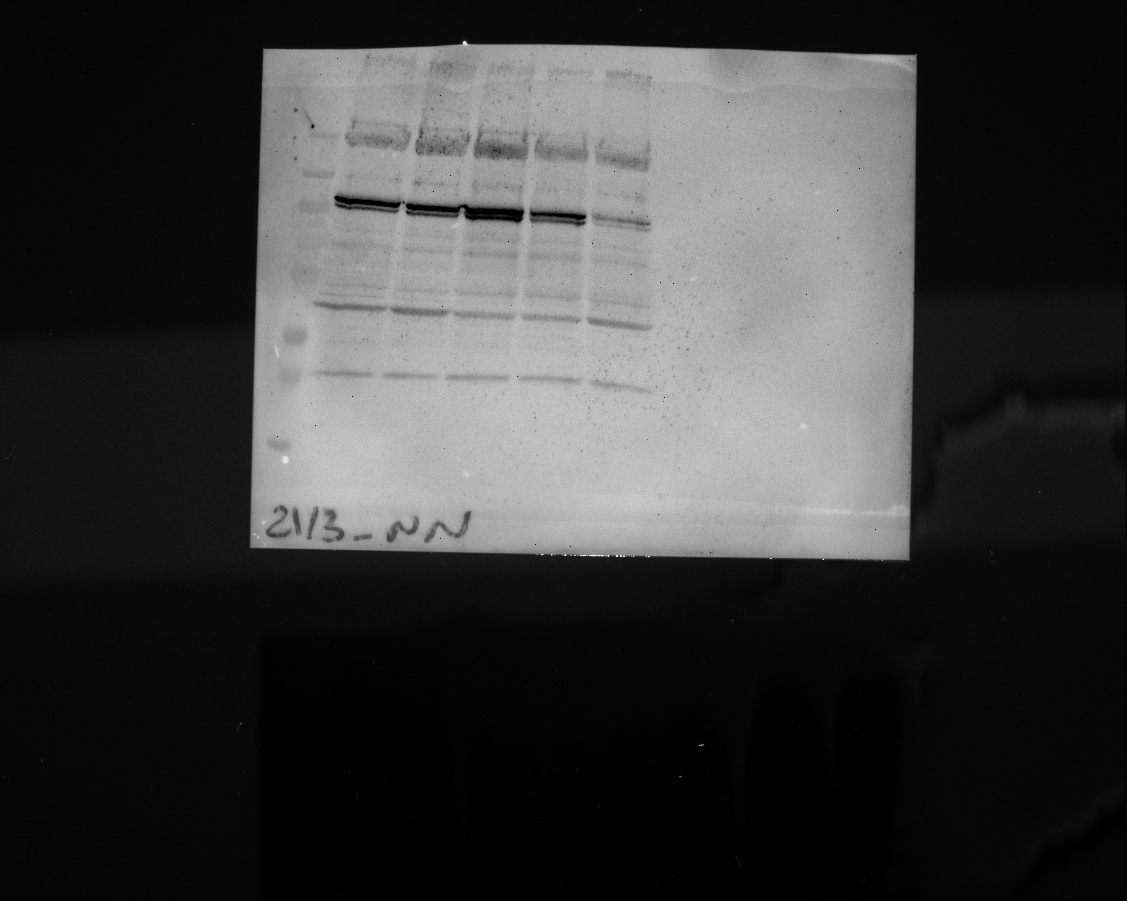

Supplement: Figure 2—source data 3. [file elife-83831-fig2-data3.zip › Figure 2_WB/Fig2a_WB/EPAC1_Fig2a.jpg]

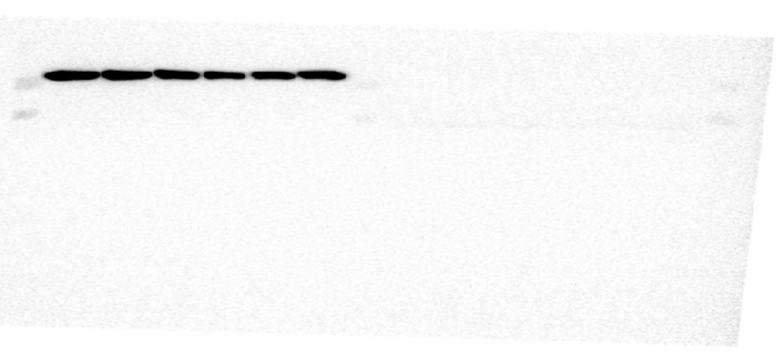

Supplement: Figure 2—source data 3. [file elife-83831-fig2-data3.zip › Figure 2_WB/Fig2b_WB/Actin_Fig2b.tif]

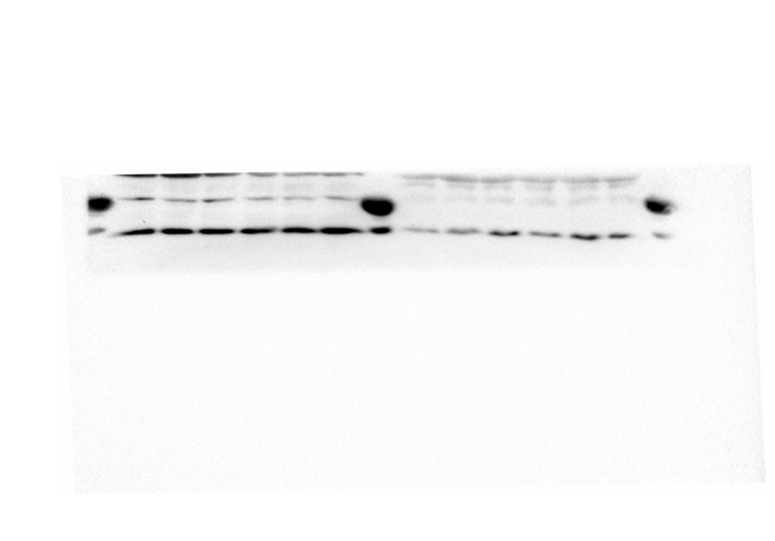

Supplement: Figure 2—source data 3. [file elife-83831-fig2-data3.zip › Figure 2_WB/Fig2b_WB/Rap1_Fig2b.tif]

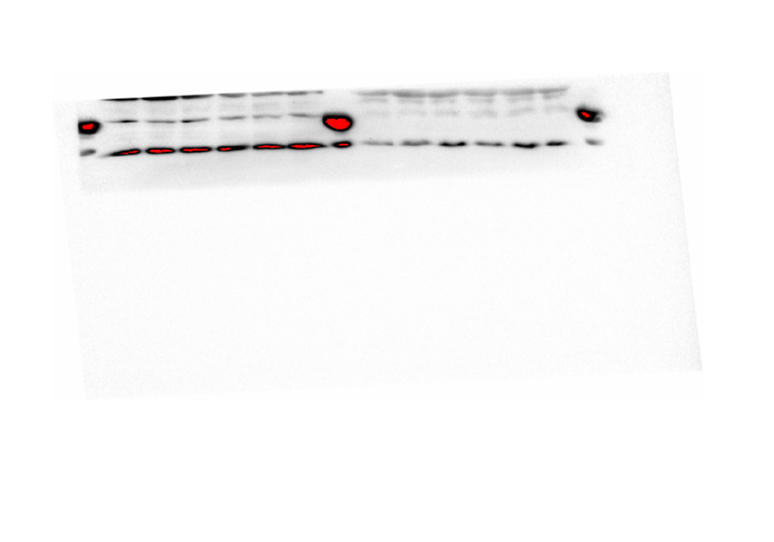

Supplement: Figure 2—source data 3. [file elife-83831-fig2-data3.zip › Figure 2_WB/Fig2b_WB/Rap1-GTP_Fig2b.tif]

Fig. 3a

Actin

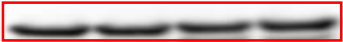

H<sub>2</sub>AX-pS139

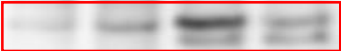

**Fig. 3b**

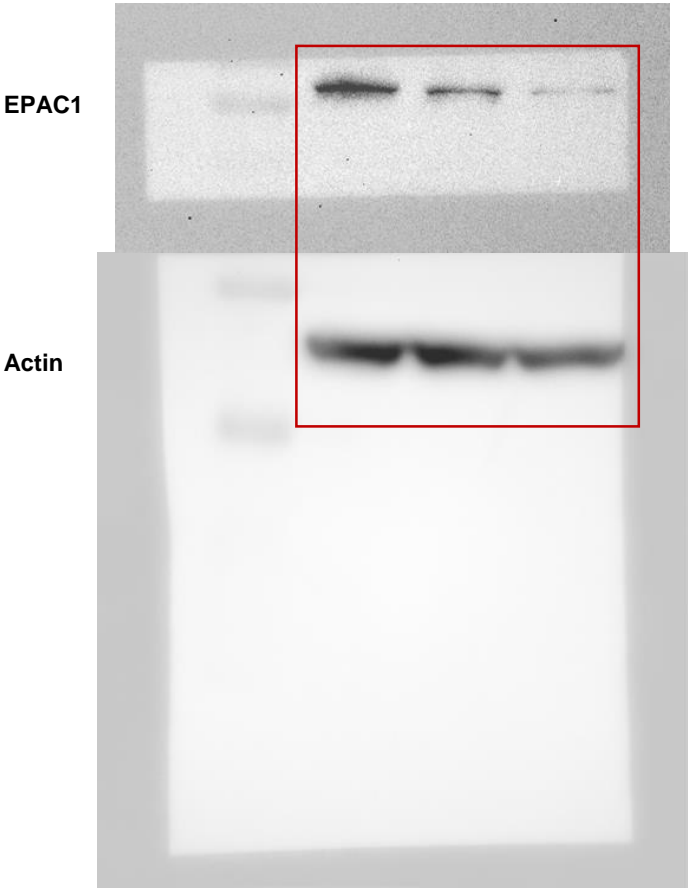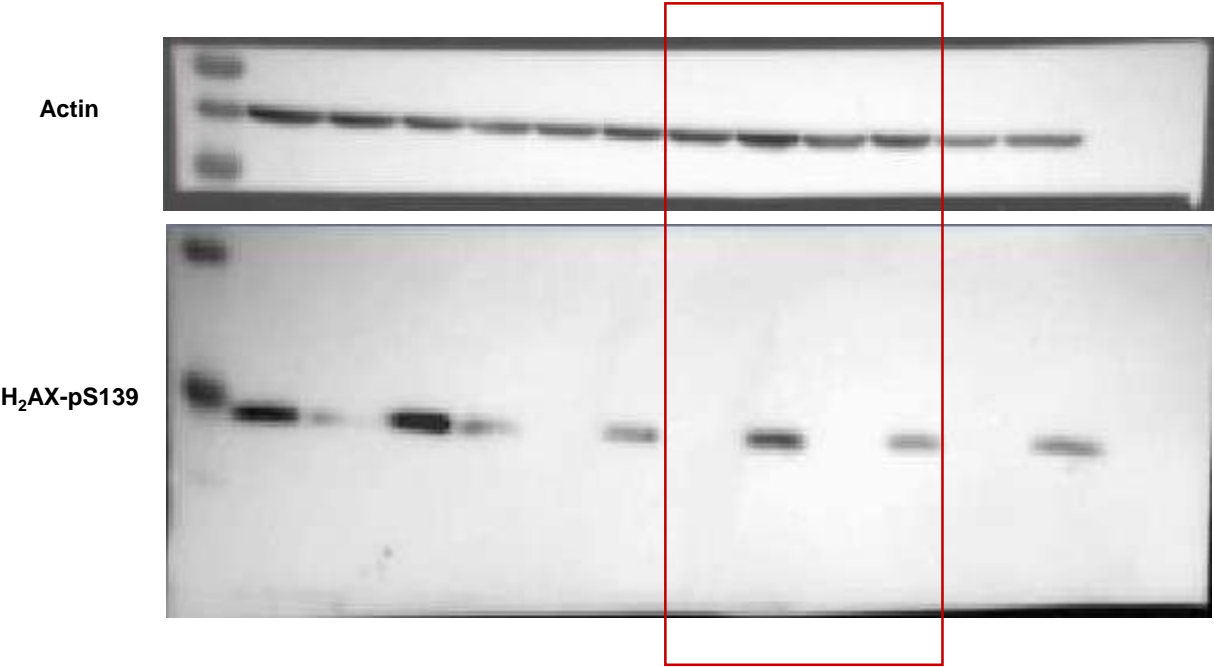

**Fig. 3c**

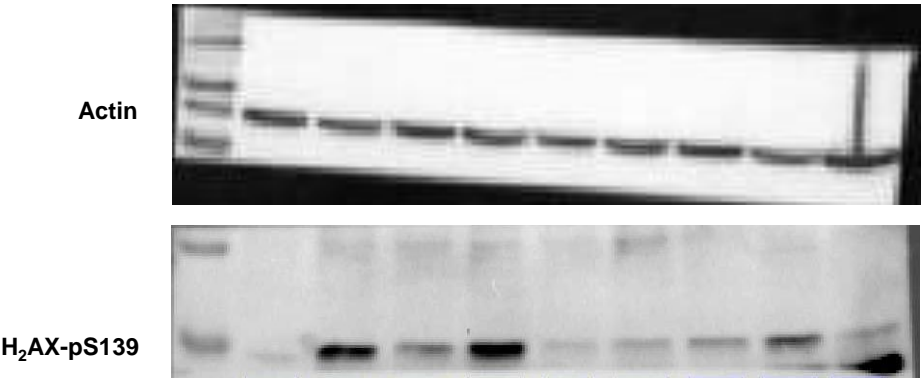

**Fig. 3d**

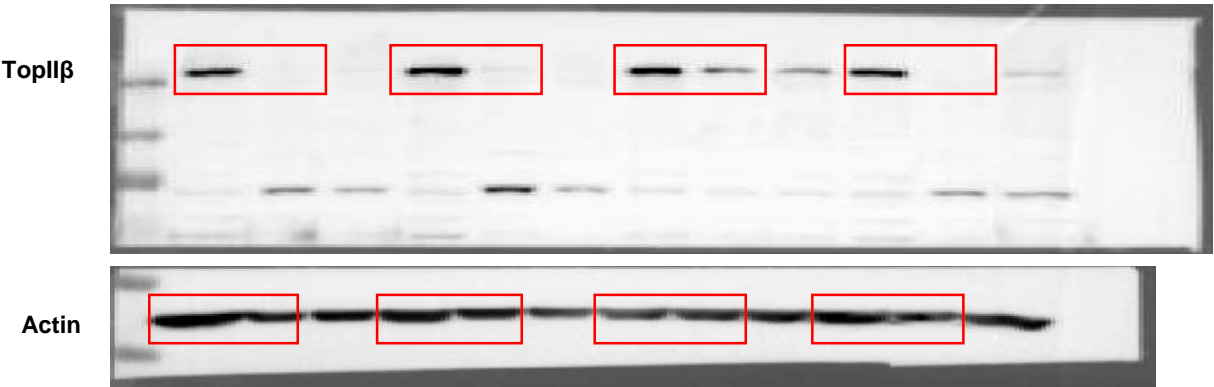

Supplement: Figure 3—source data 2. [file elife-83831-fig3-data2.zip › Data_WB_Fig3/Data_WB_Fig.3.pdf]

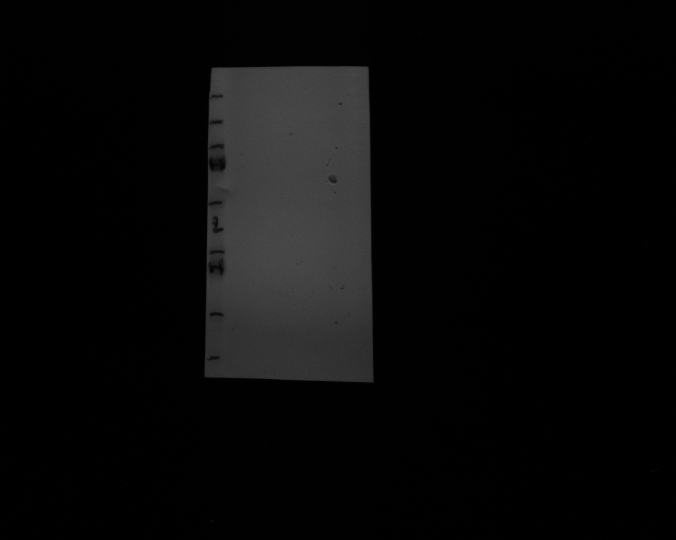

Supplement: Figure 3—source data 3. [file elife-83831-fig3-data3.zip › Figure 3_WB/Fig3a_WB/Actin_Fig3a(Membrane).tif]

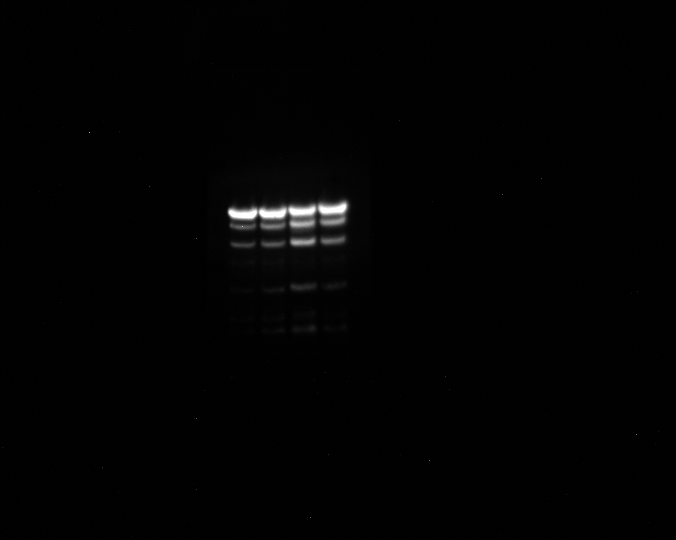

Supplement: Figure 3—source data 3. [file elife-83831-fig3-data3.zip › Figure 3_WB/Fig3a_WB/Actin_Fig3a.tif]

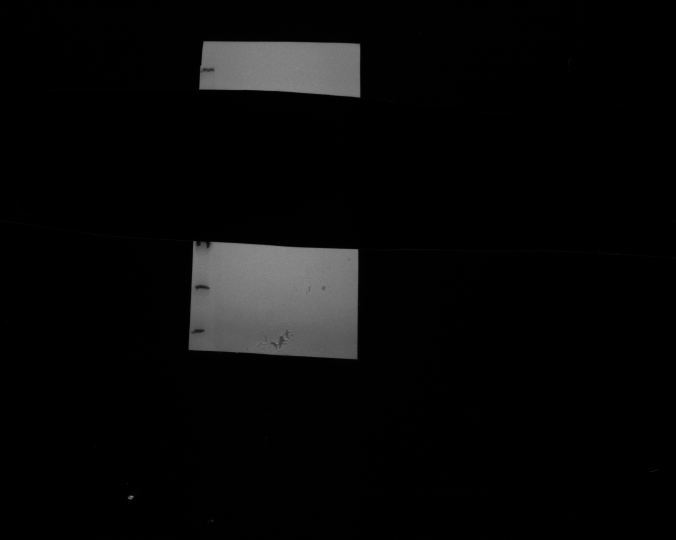

Supplement: Figure 3—source data 3. [file elife-83831-fig3-data3.zip › Figure 3_WB/Fig3a_WB/pH2AX_Fig3a(Membrane).tif]

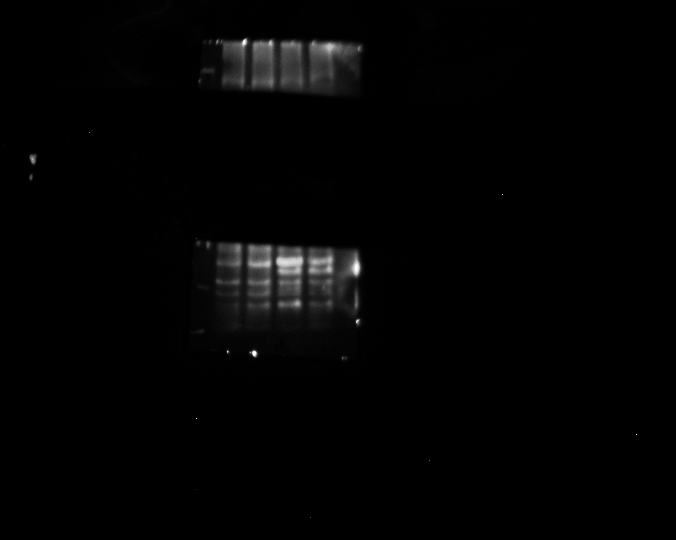

Supplement: Figure 3—source data 3. [file elife-83831-fig3-data3.zip › Figure 3_WB/Fig3a_WB/pH2AX_Fig3a.tif]

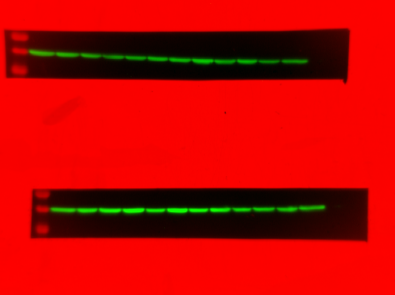

Supplement: Figure 3—source data 3. [file elife-83831-fig3-data3.zip › Figure 3_WB/Fig3b_WB/Actin_Fig3b_down.tif]

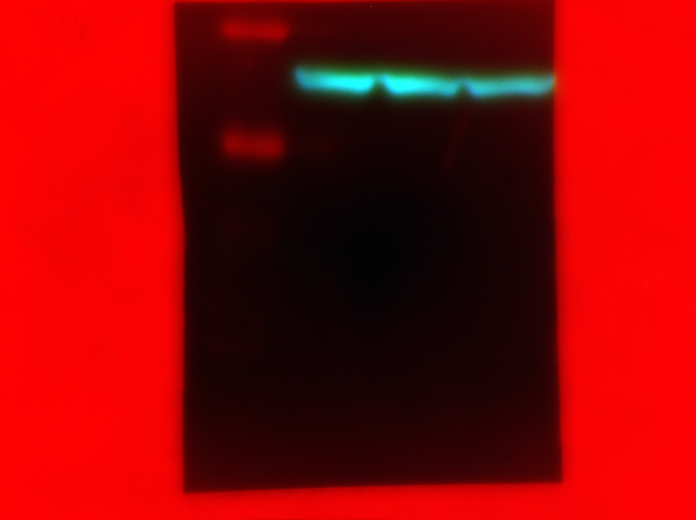

Supplement: Figure 3—source data 3. [file elife-83831-fig3-data3.zip › Figure 3_WB/Fig3b_WB/Actin_Fig3b_up.tif]

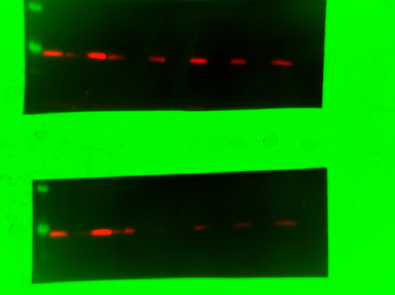

Supplement: Figure 3—source data 3. [file elife-83831-fig3-data3.zip › Figure 3_WB/Fig3b_WB/EPAC1_Fig3b_down.tif]

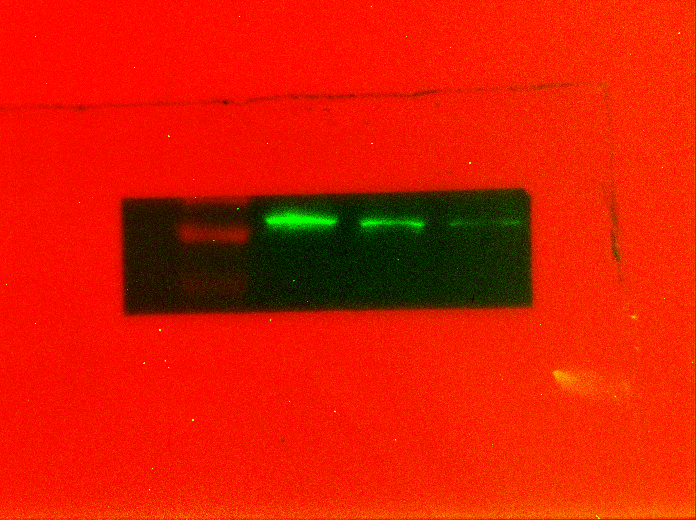

Supplement: Figure 3—source data 3. [file elife-83831-fig3-data3.zip › Figure 3_WB/Fig3b_WB/EPAC1_Fig3b_up.tif]

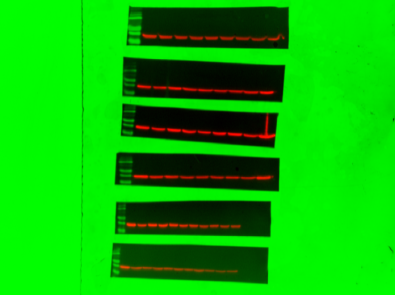

Supplement: Figure 3—source data 3. [file elife-83831-fig3-data3.zip › Figure 3_WB/Fig3c_WB/Actin_Fig3c.tif]

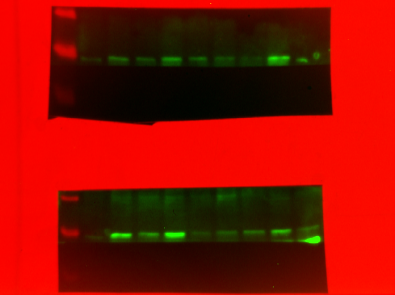

Supplement: Figure 3—source data 3. [file elife-83831-fig3-data3.zip › Figure 3_WB/Fig3c_WB/pH2AX_Fig3c.tif]

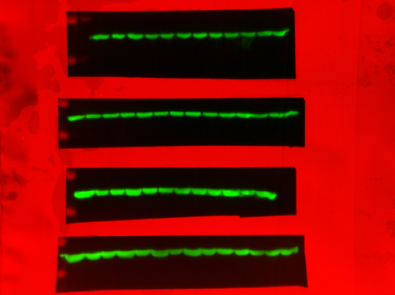

Supplement: Figure 3—source data 3. [file elife-83831-fig3-data3.zip › Figure 3_WB/Fig3d_WB/Actin_Fig3d.tif]

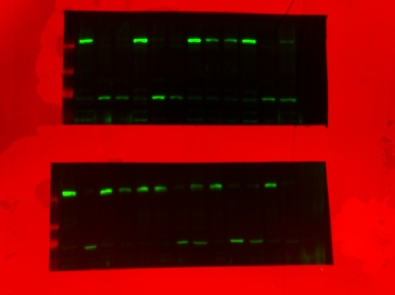

Supplement: Figure 3—source data 3. [file elife-83831-fig3-data3.zip › Figure 3_WB/Fig3d_WB/TopII_Fig3d.tif]

**Fig. 4e**

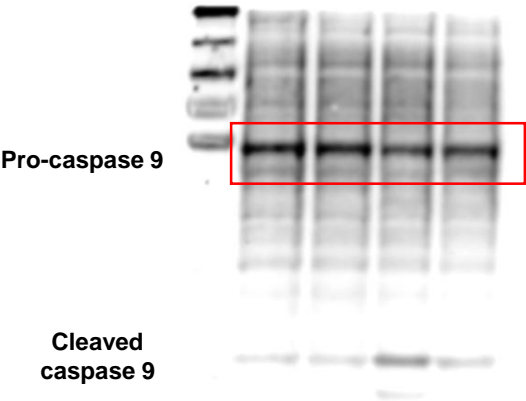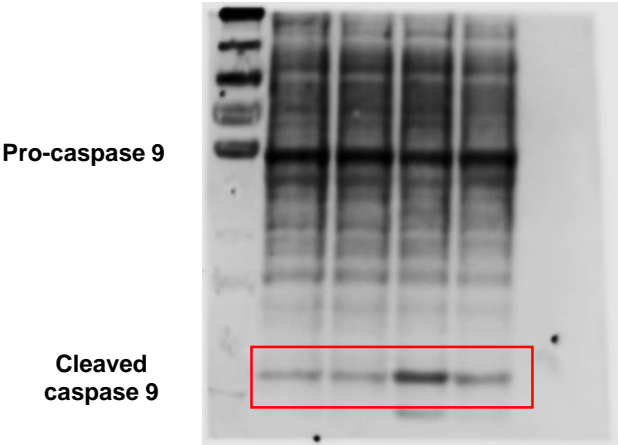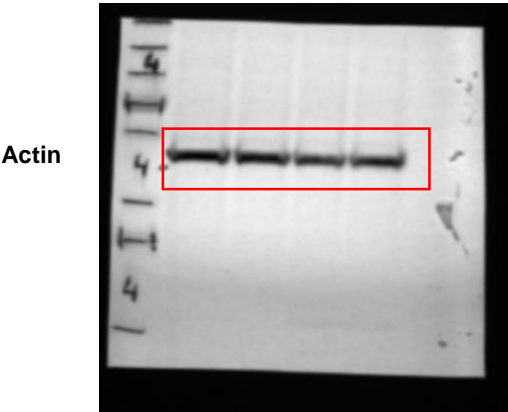

**Fig. 4f**

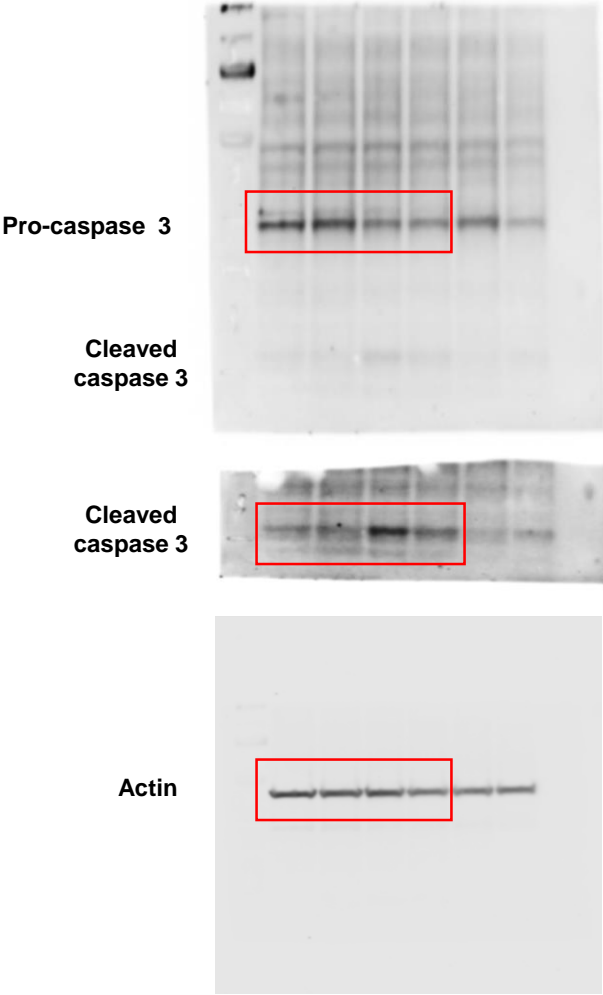

Supplement: Figure 4—source data 2. [file elife-83831-fig4-data2.zip › Data_WB_Fig4/Data_WB_Fig.4.pdf]

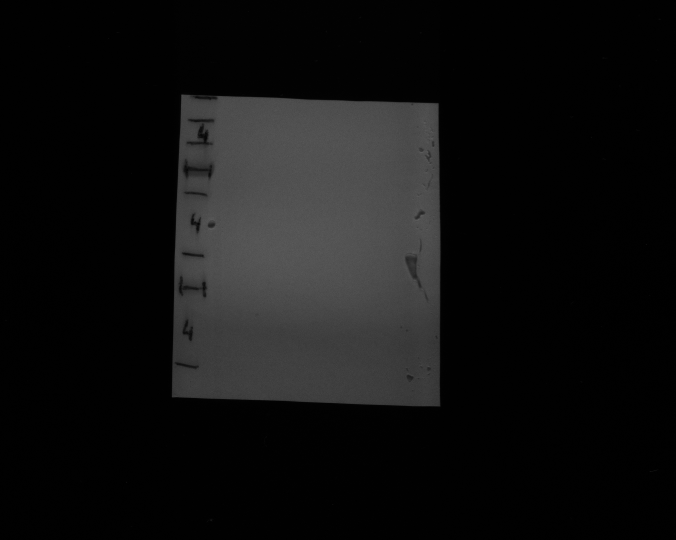

Supplement: Figure 4—source data 3. [file elife-83831-fig4-data3.zip › Figure 4_WB/Fig4e_WB/Actin_Fig4e(Membrane).tif]

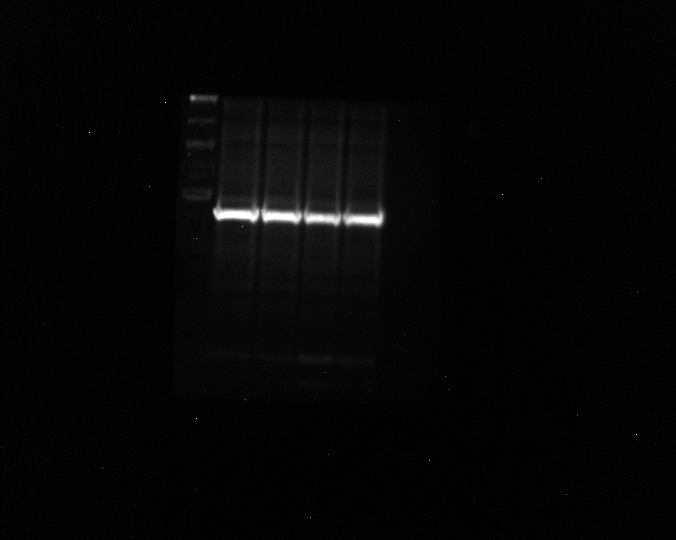

Supplement: Figure 4—source data 3. [file elife-83831-fig4-data3.zip › Figure 4_WB/Fig4e_WB/Actin_Fig4e.tif]

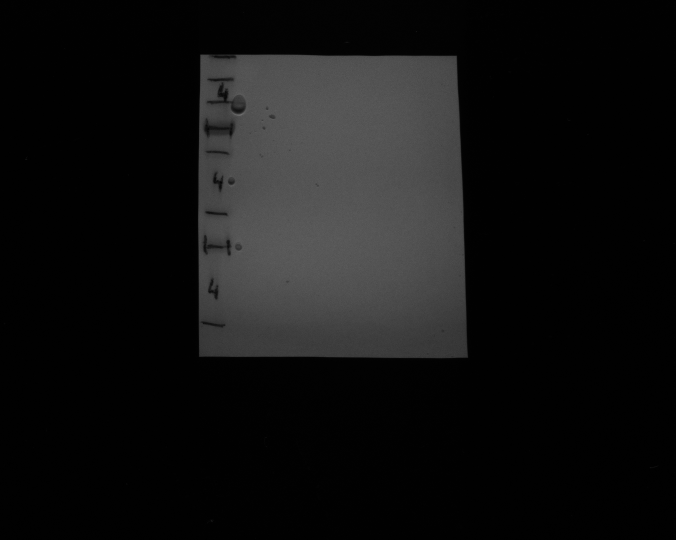

Supplement: Figure 4—source data 3. [file elife-83831-fig4-data3.zip › Figure 4_WB/Fig4e_WB/Caspase9_Fig4e(Membrane).tif]

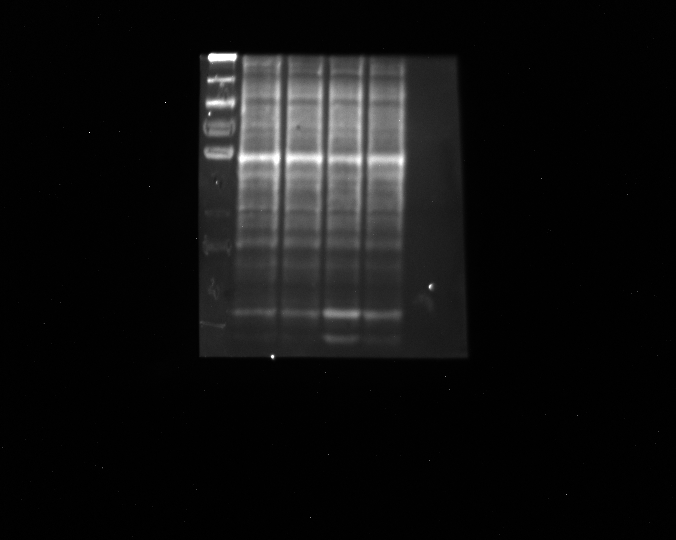

Supplement: Figure 4—source data 3. [file elife-83831-fig4-data3.zip › Figure 4_WB/Fig4e_WB/Caspase9_Fig4e.tif]

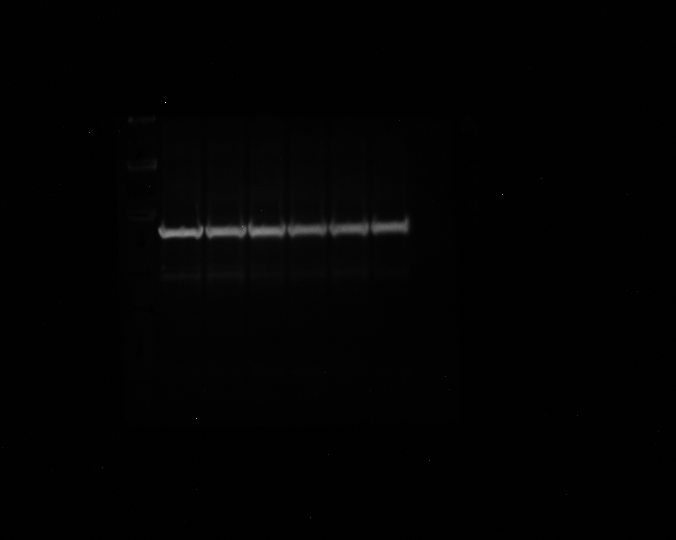

Supplement: Figure 4—source data 3. [file elife-83831-fig4-data3.zip › Figure 4_WB/Fig4f_WB/Actin_Fig4f.tif]

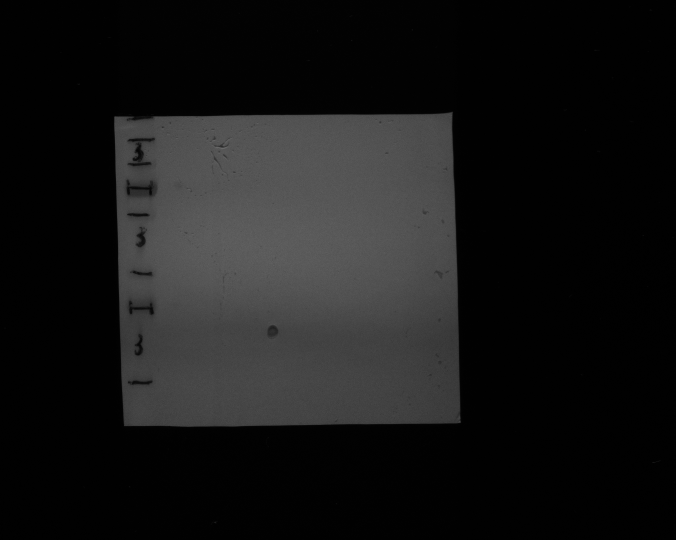

Supplement: Figure 4—source data 3. [file elife-83831-fig4-data3.zip › Figure 4_WB/Fig4f_WB/Actine_Fig4f(Membrane).tif]

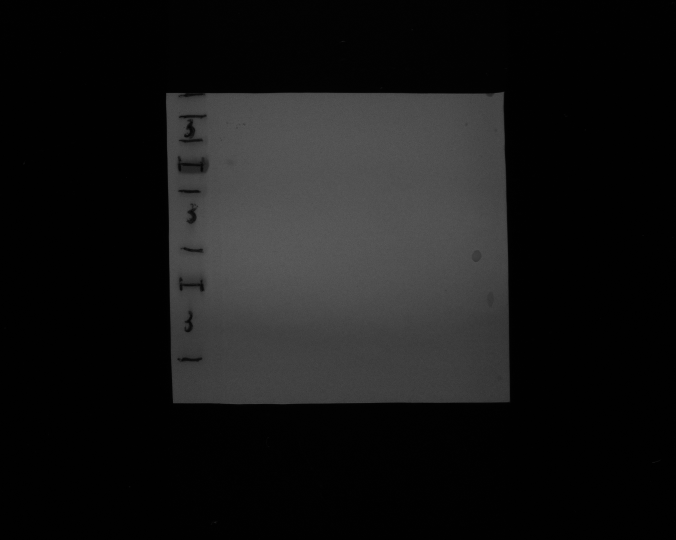

Supplement: Figure 4—source data 3. [file elife-83831-fig4-data3.zip › Figure 4_WB/Fig4f_WB/Caspase3_Fig4f(Membrane).tif]

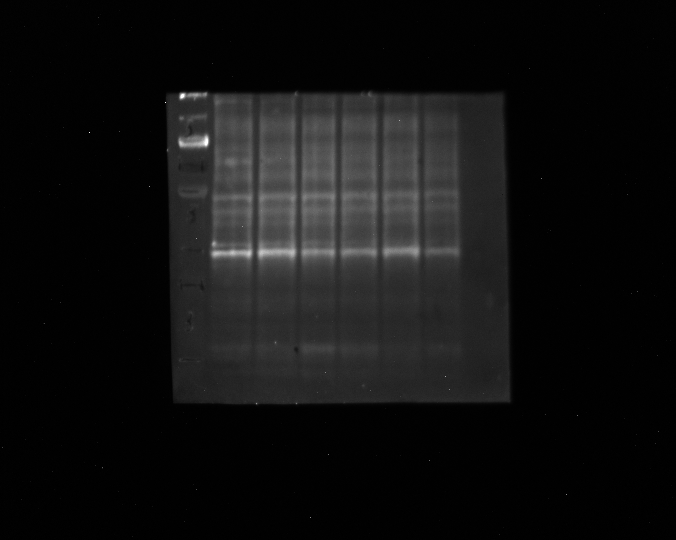

Supplement: Figure 4—source data 3. [file elife-83831-fig4-data3.zip › Figure 4_WB/Fig4f_WB/Caspase3_Fig4f.tif]

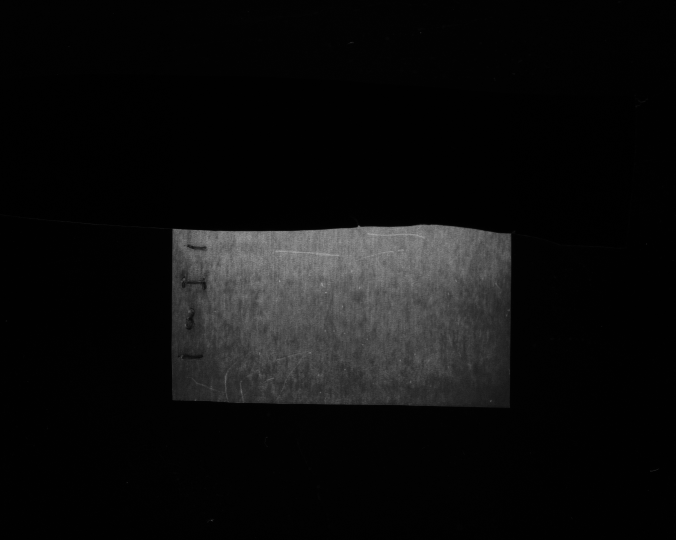

Supplement: Figure 4—source data 3. [file elife-83831-fig4-data3.zip › Figure 4_WB/Fig4f_WB/Cleaved-Caspase3_Fig4f(Membrane).tif]

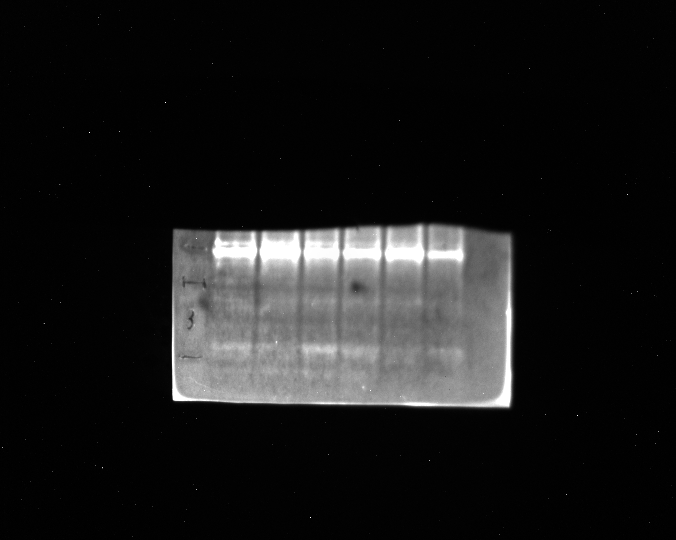

Supplement: Figure 4—source data 3. [file elife-83831-fig4-data3.zip › Figure 4_WB/Fig4f_WB/Cleaved-Caspase3_Fig4f.tif]

# Fig5d 2w

Raw data

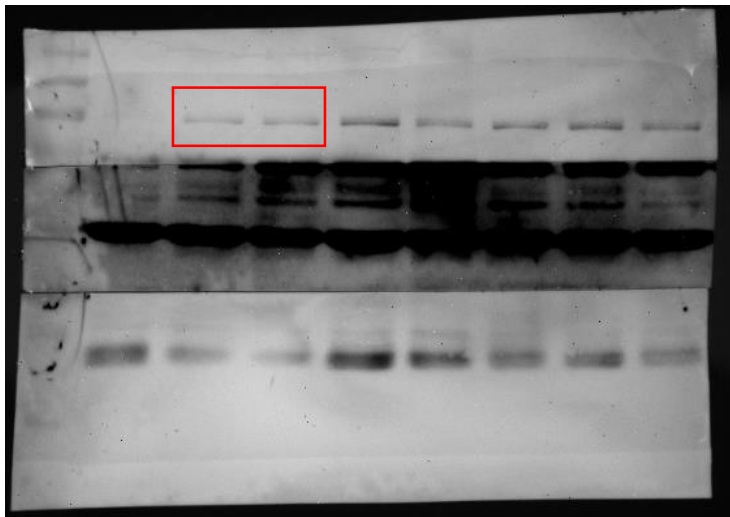

Epac1

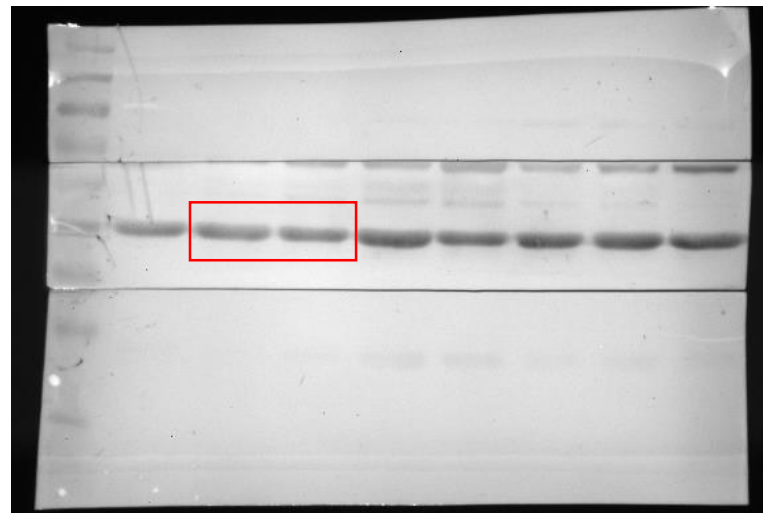

Actin

# Fig5d 6w

Raw data

Epac1

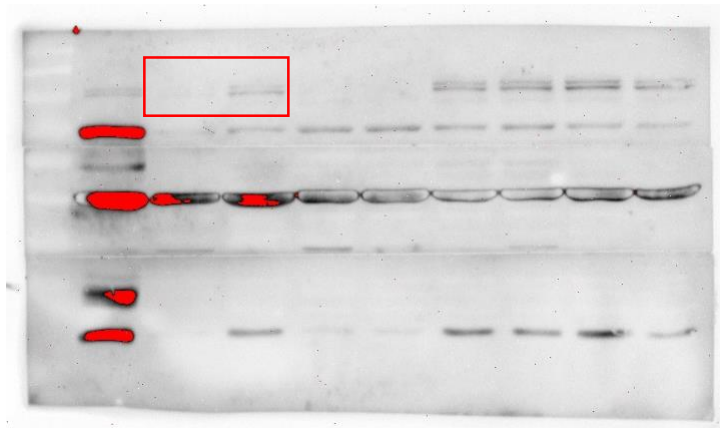

Actin

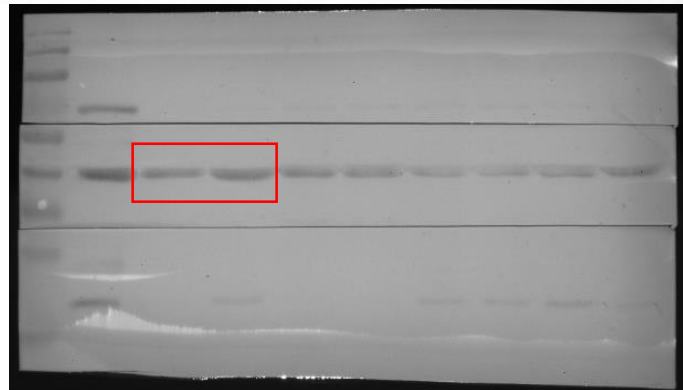

# Fig5d 15w

Raw data

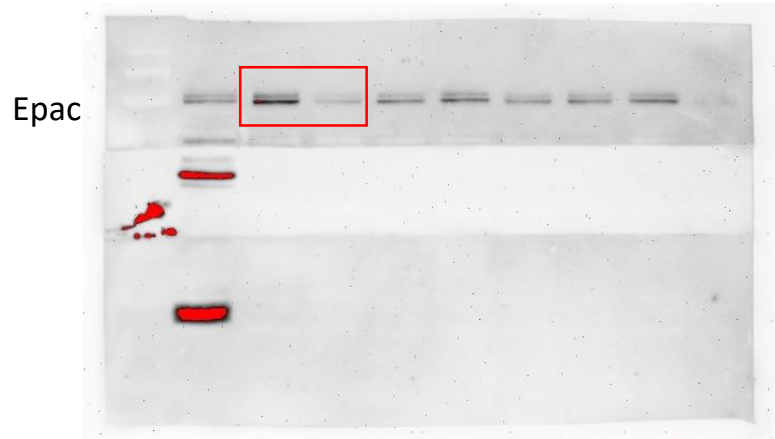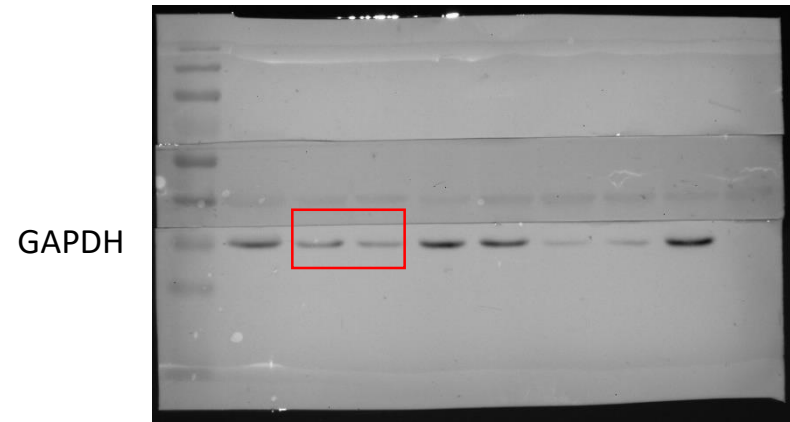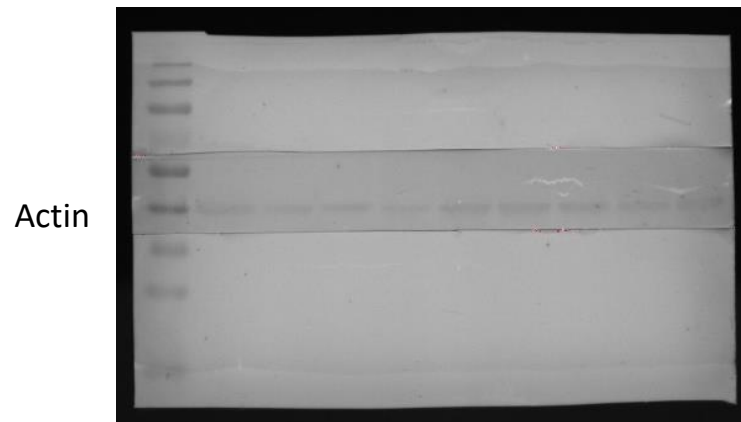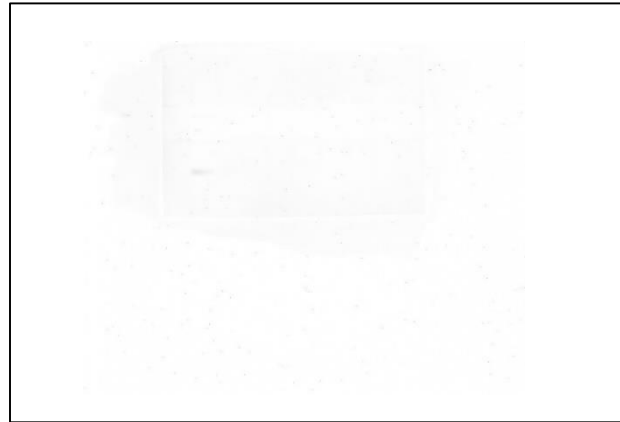

Supplement: Figure 5—source data 2. [file elife-83831-fig5-data2.zip › Data_WB_Fig5/App_Fig5d.pdf]

Fig. 5d

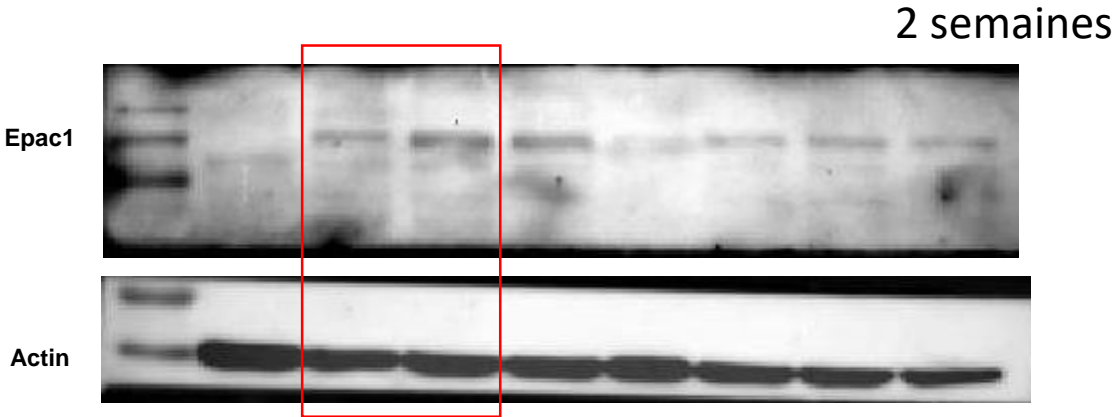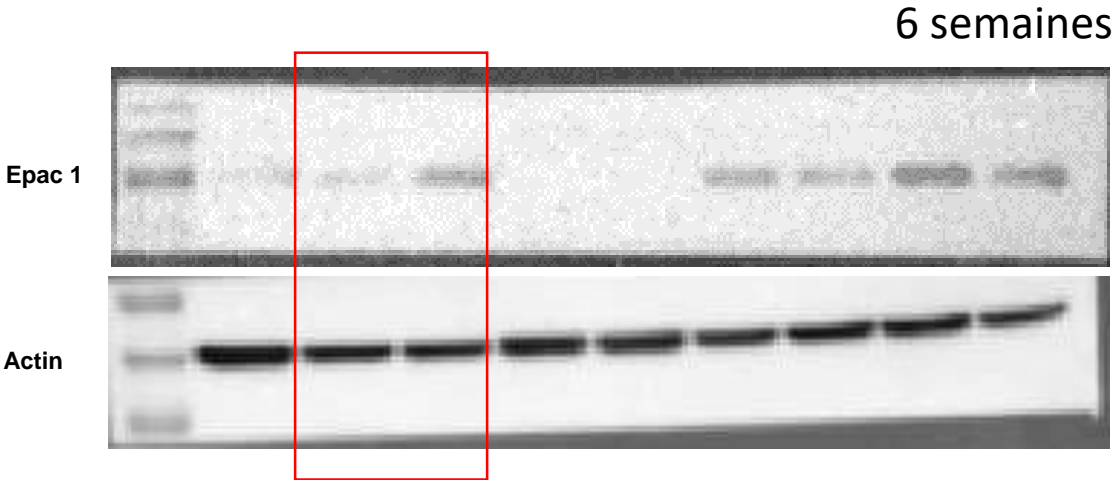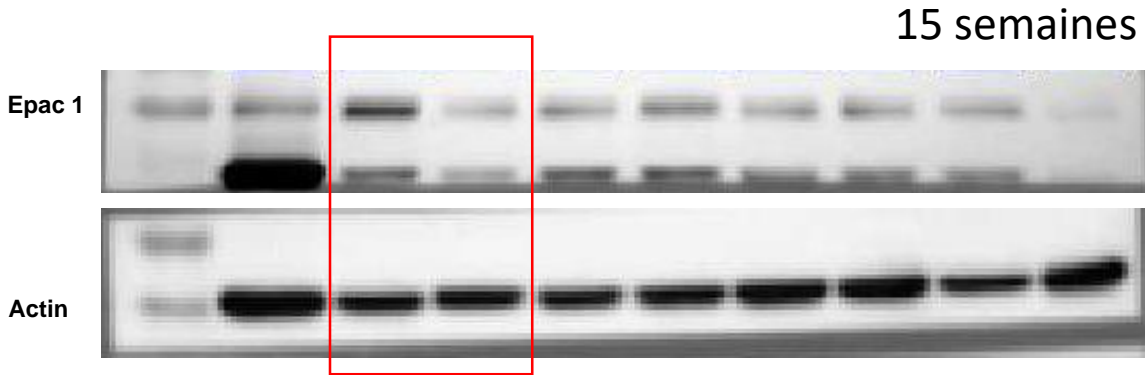

**Fig. 5e**

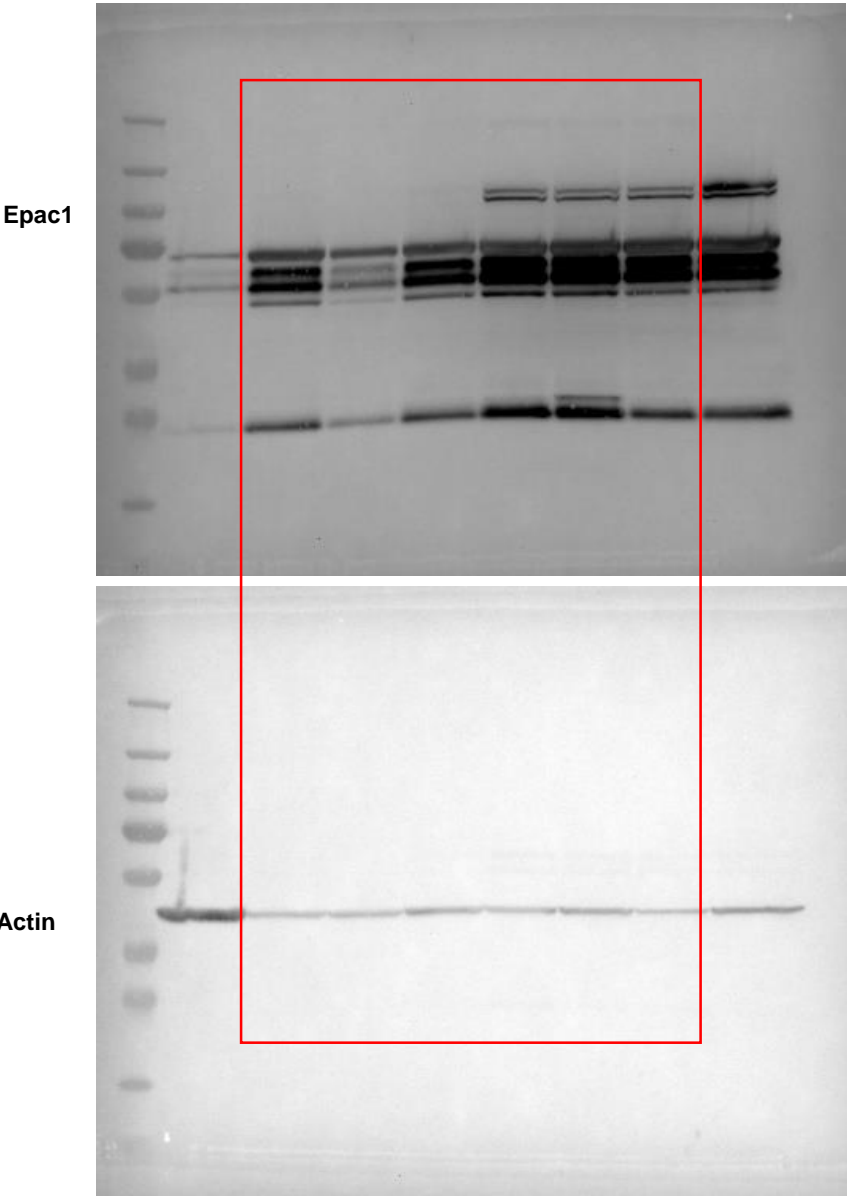

Supplement: Figure 5—source data 2. [file elife-83831-fig5-data2.zip › Data_WB_Fig5/Data_WB_Fig.5.pdf]

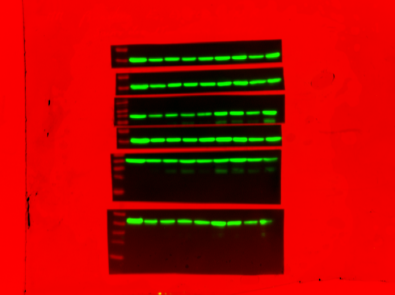

Supplement: Figure 5—source data 3. [file elife-83831-fig5-data3.zip › Figure 5_WB/Fig5d_WB/Actin_15weeks_Fig5d.tif]

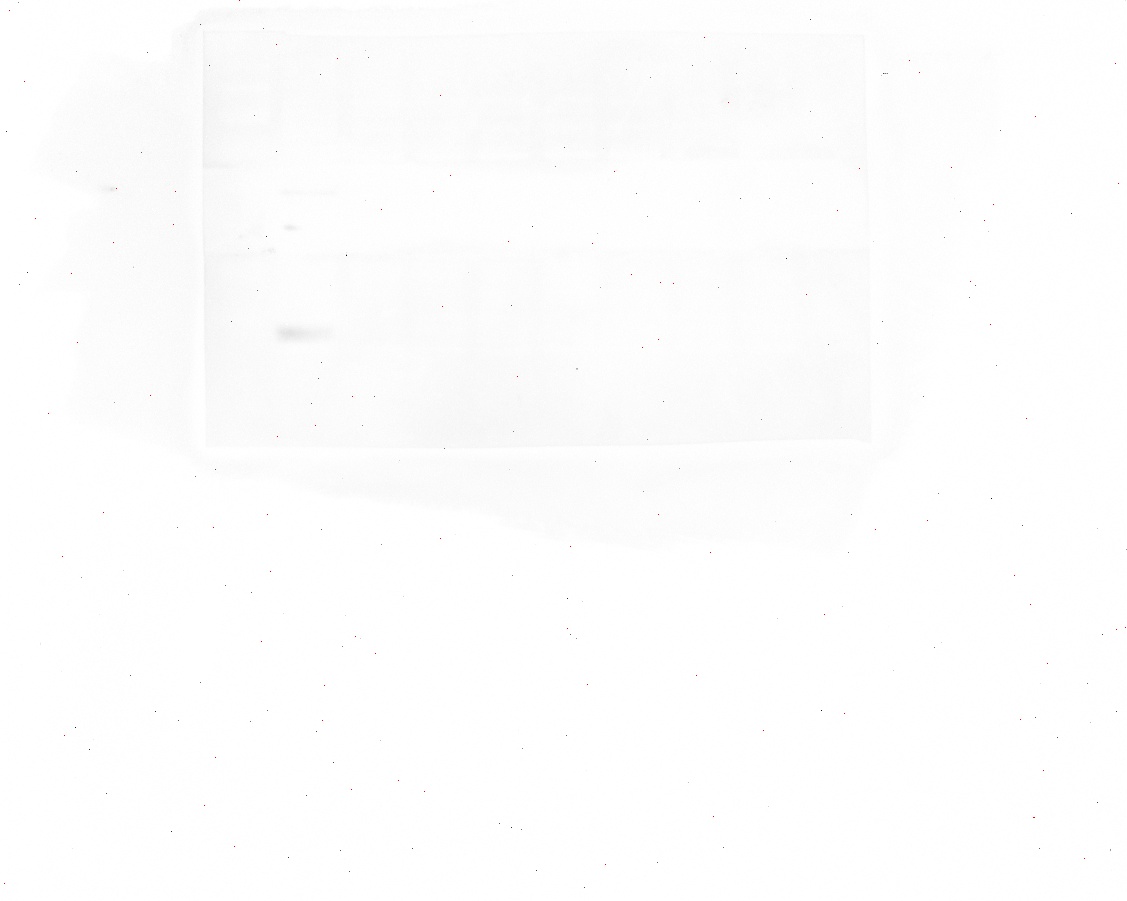

Supplement: Figure 5—source data 3. [file elife-83831-fig5-data3.zip › Figure 5_WB/Fig5d_WB/Fig5d_15w_Actin(Chemi).jpg]

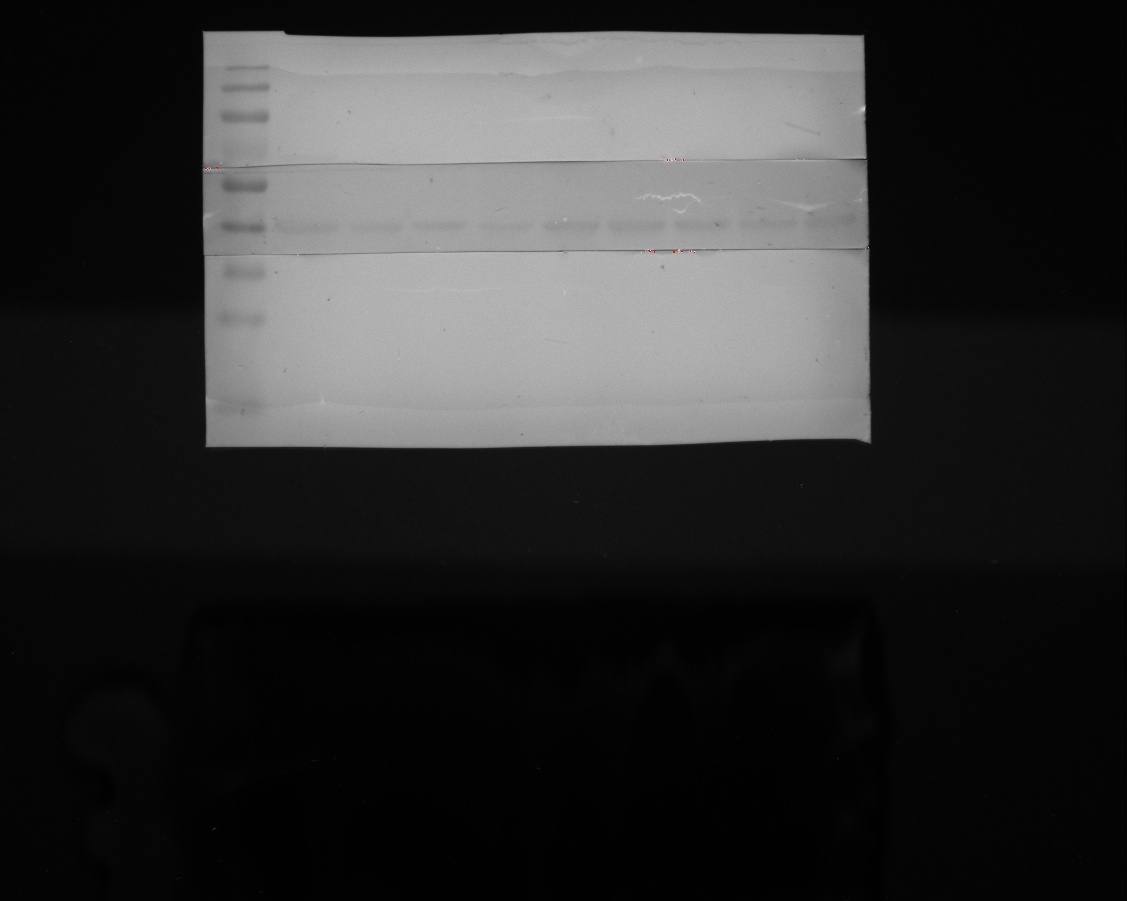

Supplement: Figure 5—source data 3. [file elife-83831-fig5-data3.zip › Figure 5_WB/Fig5d_WB/Fig5d_15w_Actin(Membrane).jpg]

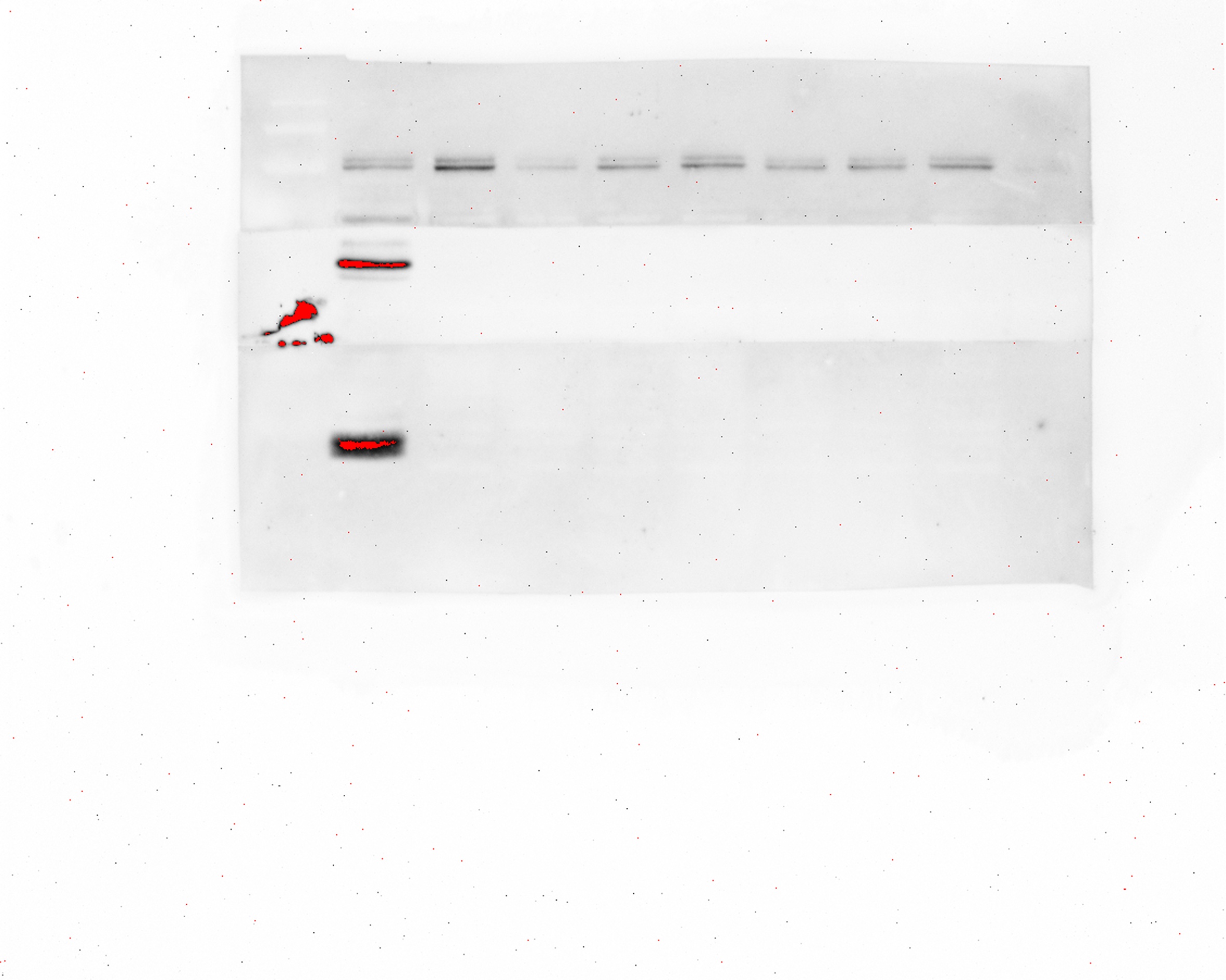

Supplement: Figure 5—source data 3. [file elife-83831-fig5-data3.zip › Figure 5_WB/Fig5d_WB/Fig5d_15w_EPAC1.jpg]

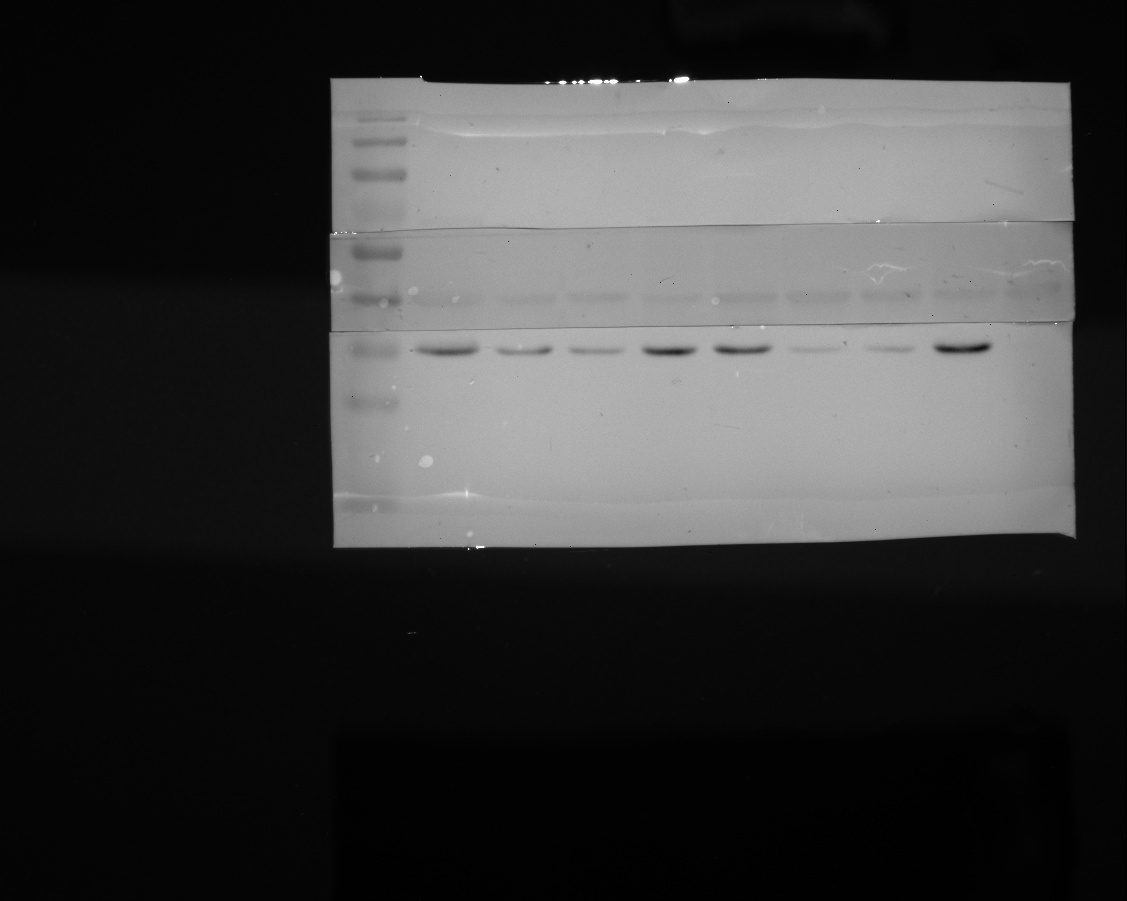

Supplement: Figure 5—source data 3. [file elife-83831-fig5-data3.zip › Figure 5_WB/Fig5d_WB/Fig5d_15w_GAPDH.jpg]

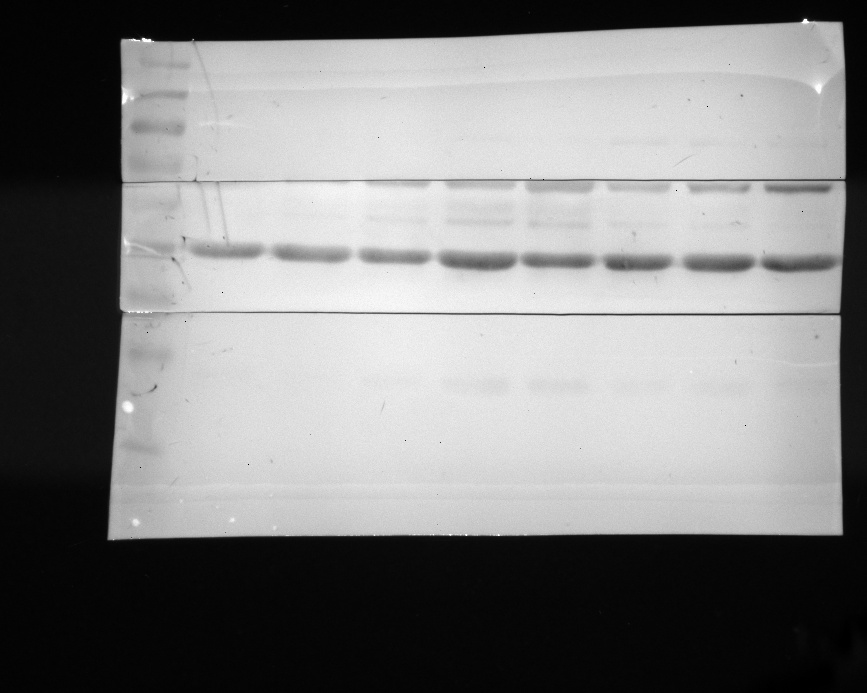

Supplement: Figure 5—source data 3. [file elife-83831-fig5-data3.zip › Figure 5_WB/Fig5d_WB/Fig5d_2w_Actin.jpg]

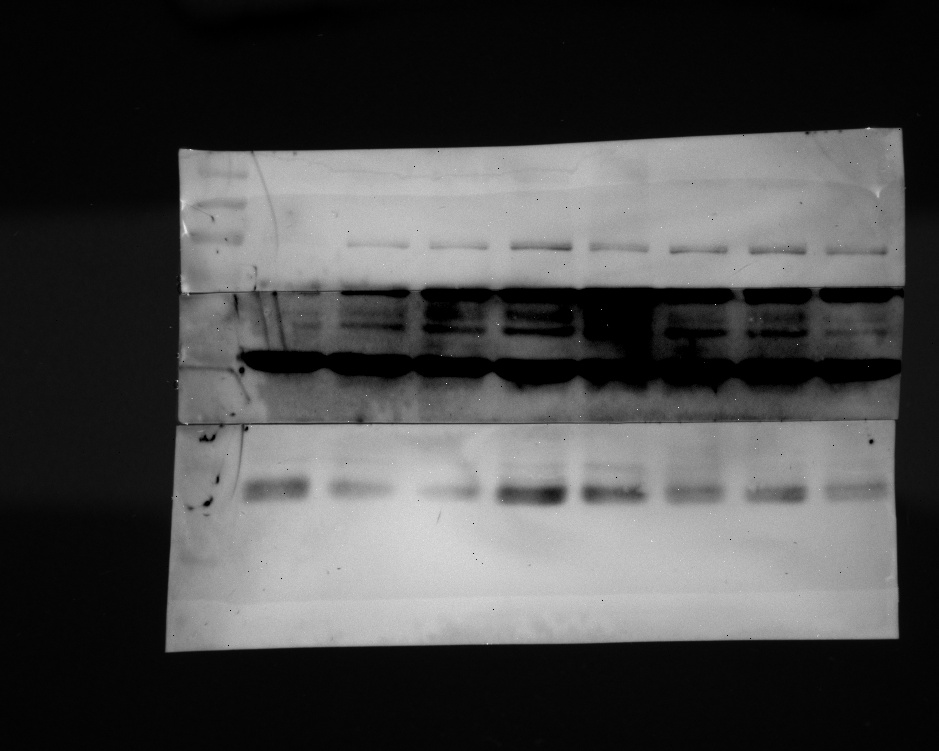

Supplement: Figure 5—source data 3. [file elife-83831-fig5-data3.zip › Figure 5_WB/Fig5d_WB/Fig5d_2w_EPAC1.jpg]

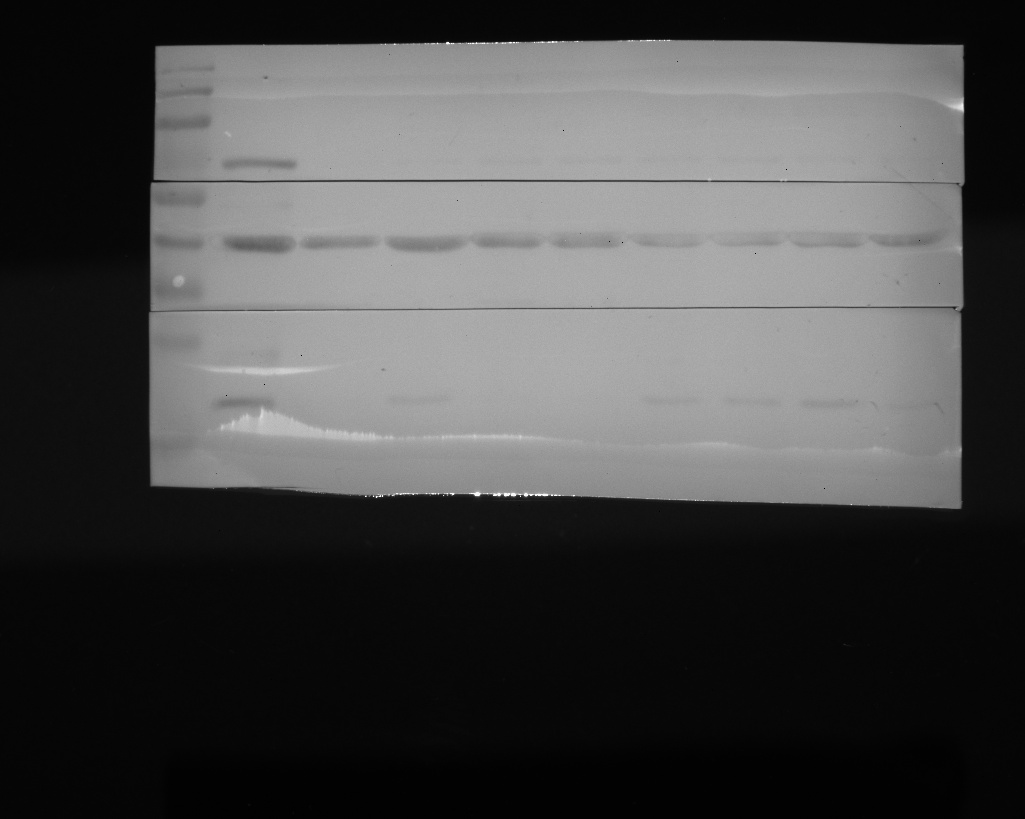

Supplement: Figure 5—source data 3. [file elife-83831-fig5-data3.zip › Figure 5_WB/Fig5d_WB/Fig5d_6w_Actin.jpg]

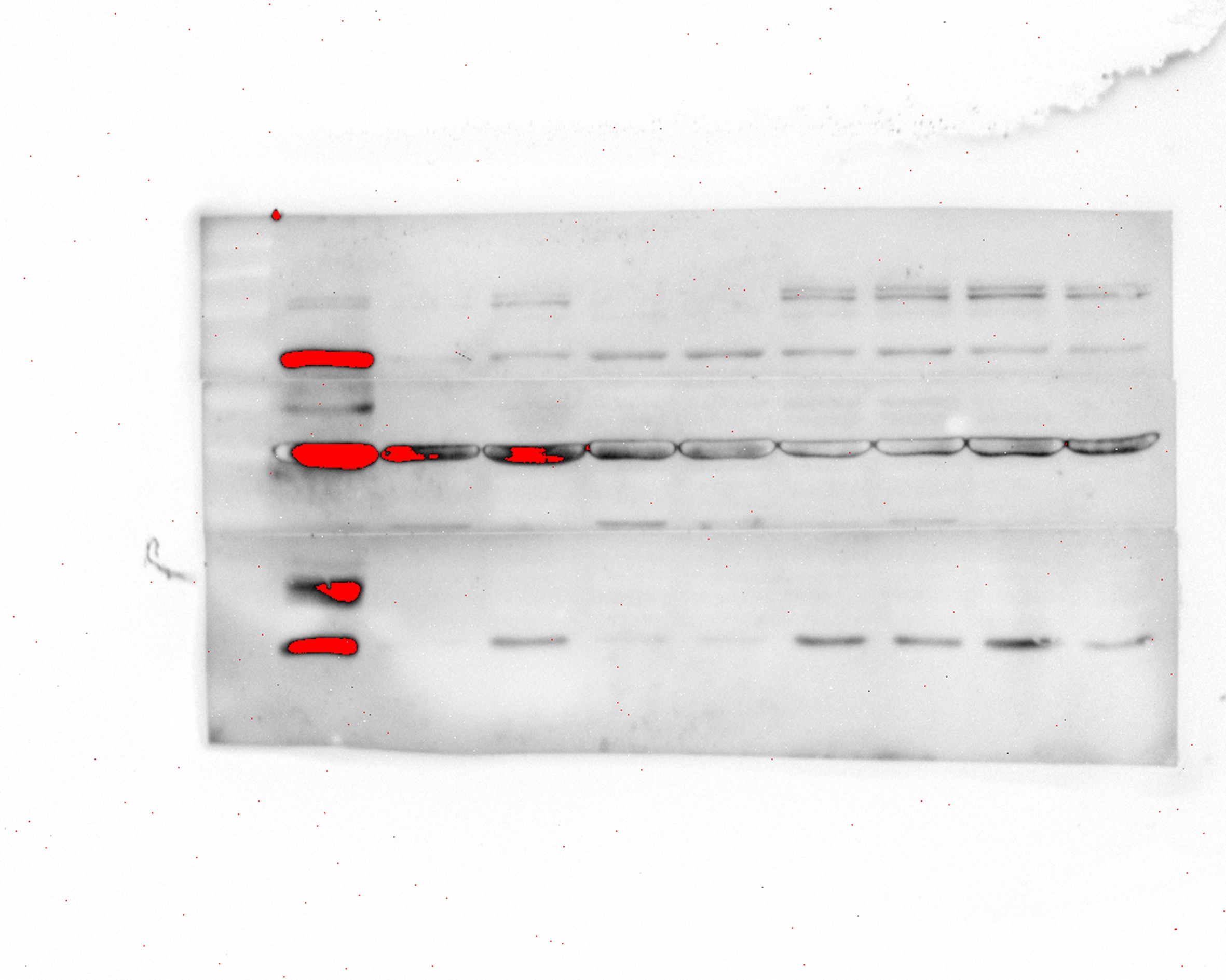

Supplement: Figure 5—source data 3. [file elife-83831-fig5-data3.zip › Figure 5_WB/Fig5d_WB/Fig5d_6w_EPAC1.jpg]

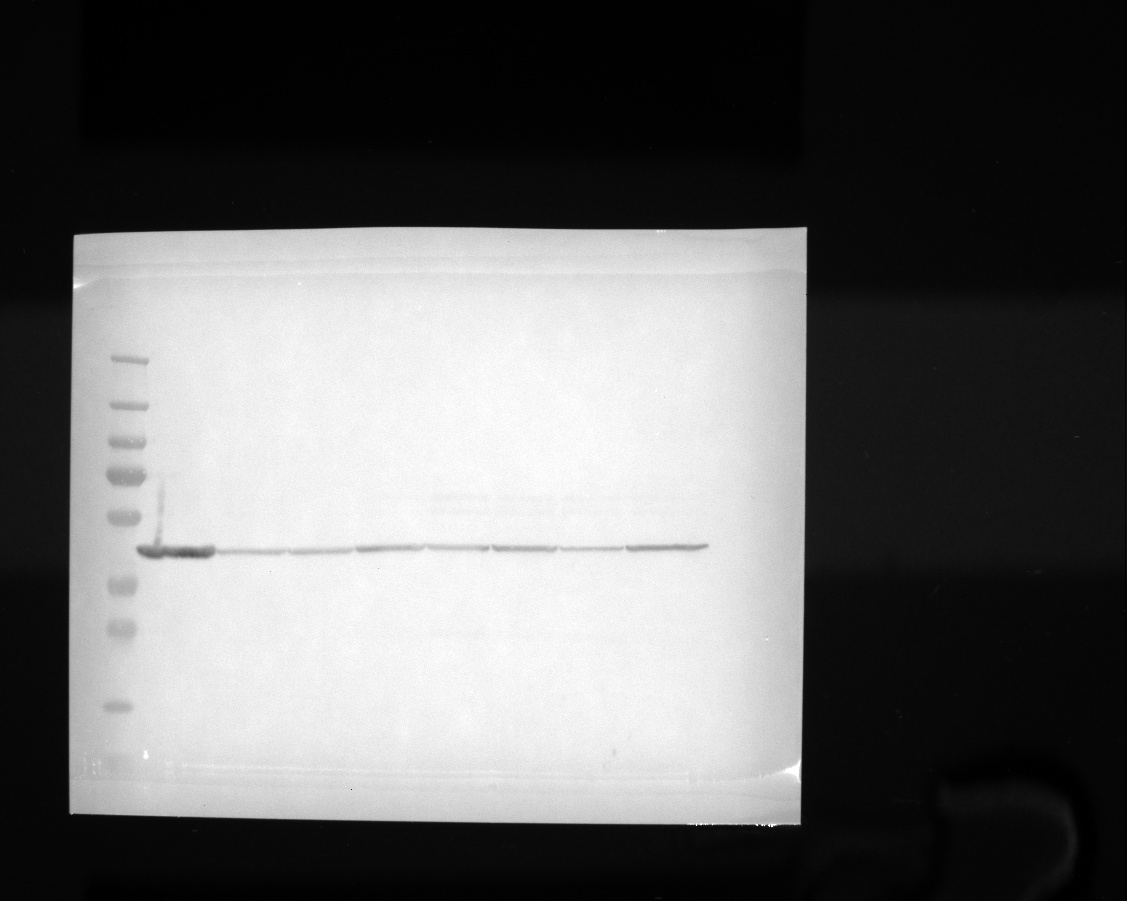

Supplement: Figure 5—source data 3. [file elife-83831-fig5-data3.zip › Figure 5_WB/Fig5e_WB/Fig5e_Actin.jpg]

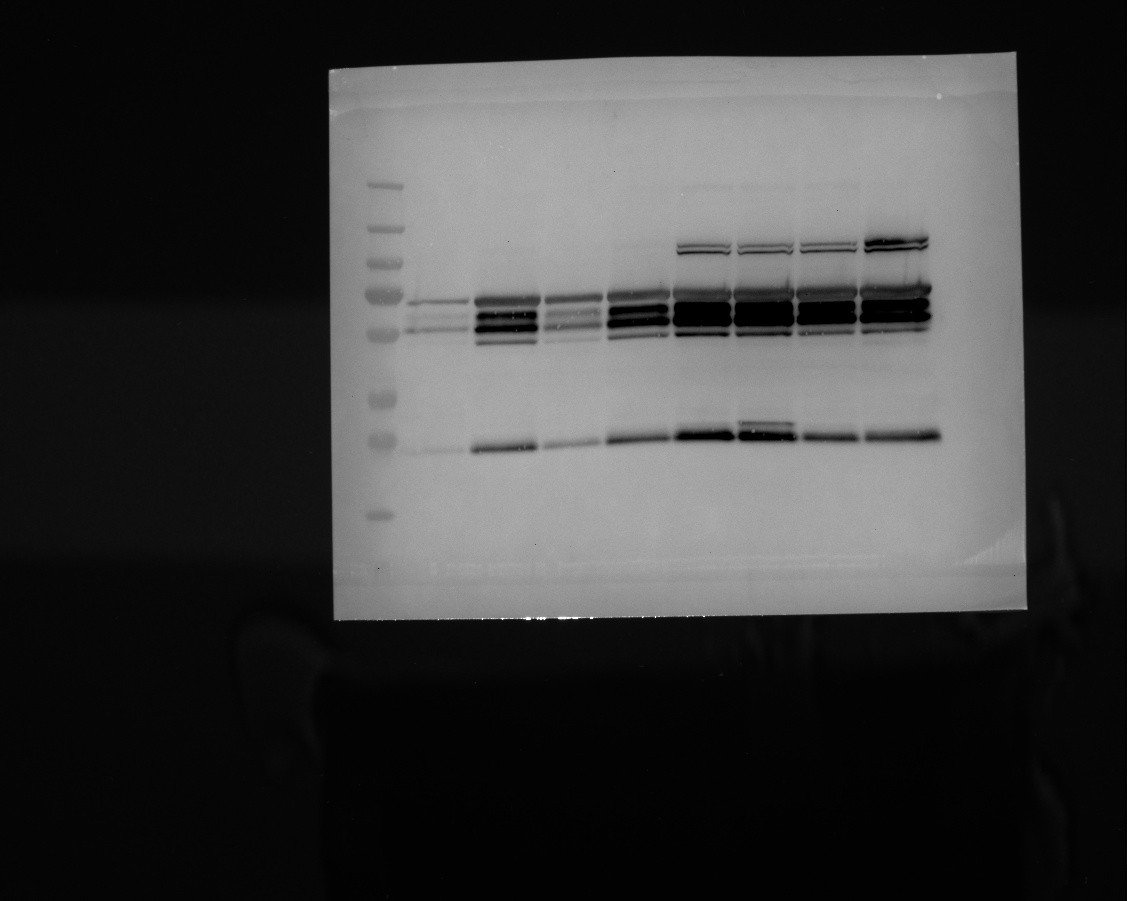

Supplement: Figure 5—source data 3. [file elife-83831-fig5-data3.zip › Figure 5_WB/Fig5e_WB/Fig5e_Epac1.jpg]

# Fig6d

Raw data

WT

Serca2

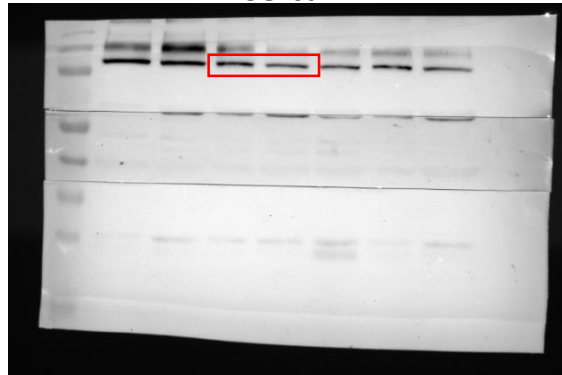

Calsequestrin

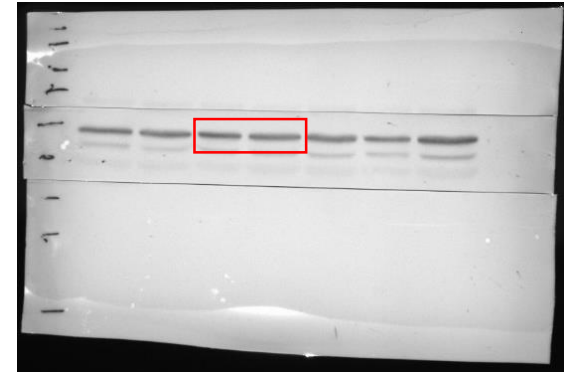

EPAC1 KO

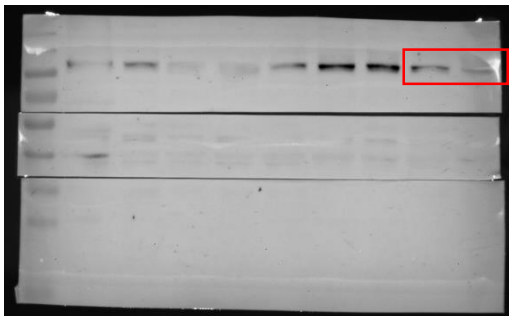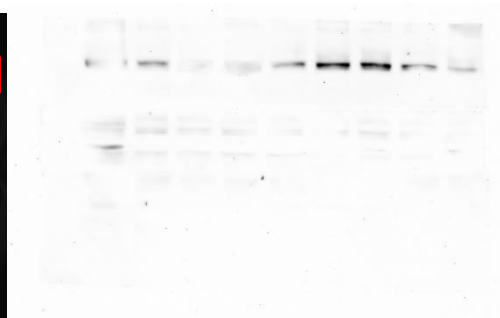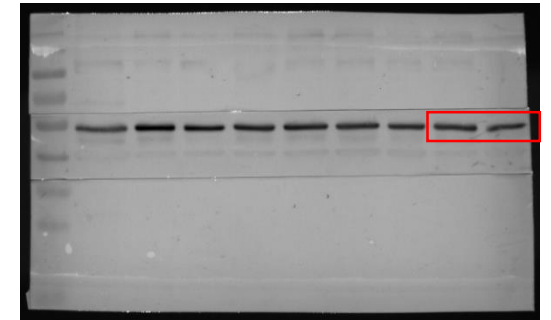

# Fig6d

Raw data\_LICOR (total protein) staining

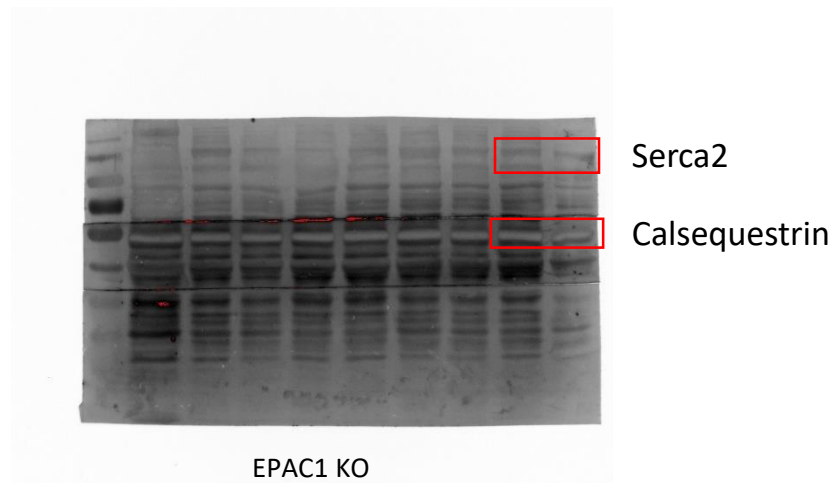

Supplement: Figure 6—source data 2. [file elife-83831-fig6-data2.zip › Data_WB_Fig6/App_Fig6d.pdf]

Fig. 6d

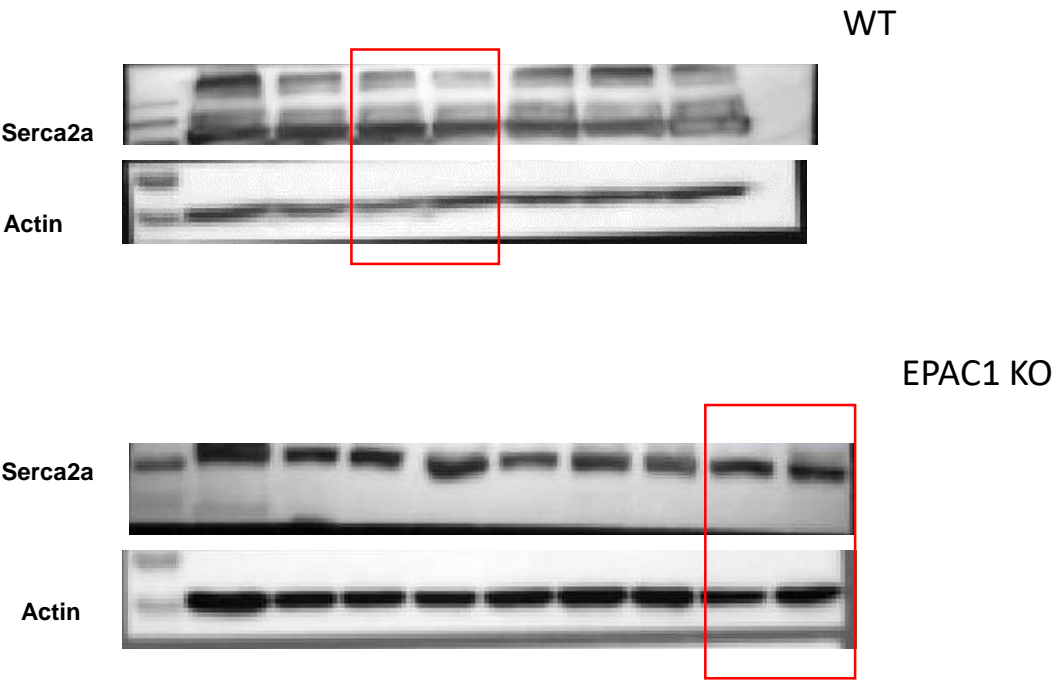

Supplement: Figure 6—source data 2. [file elife-83831-fig6-data2.zip › Data_WB_Fig6/Data_WB_Fig.6.pdf]

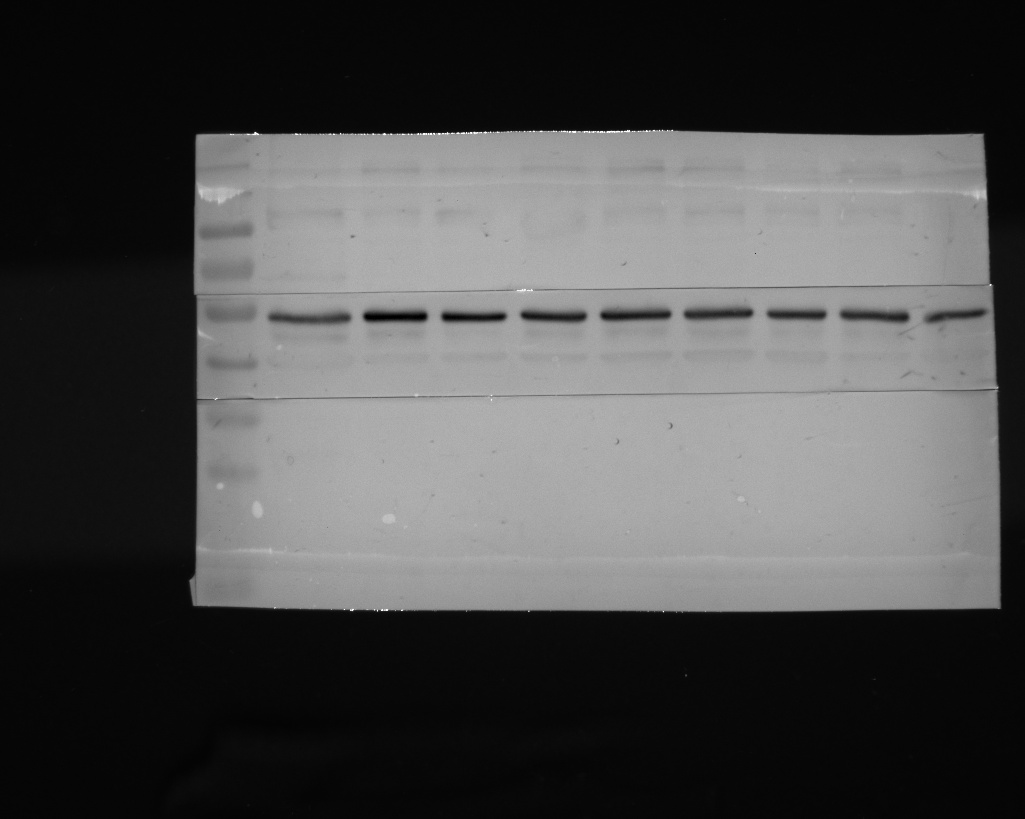

Supplement: Figure 6—source data 3. [file elife-83831-fig6-data3.zip › Figure 6_WB/Fig6d_WB/Fig6d_KO_Calsequestrin.jpg]

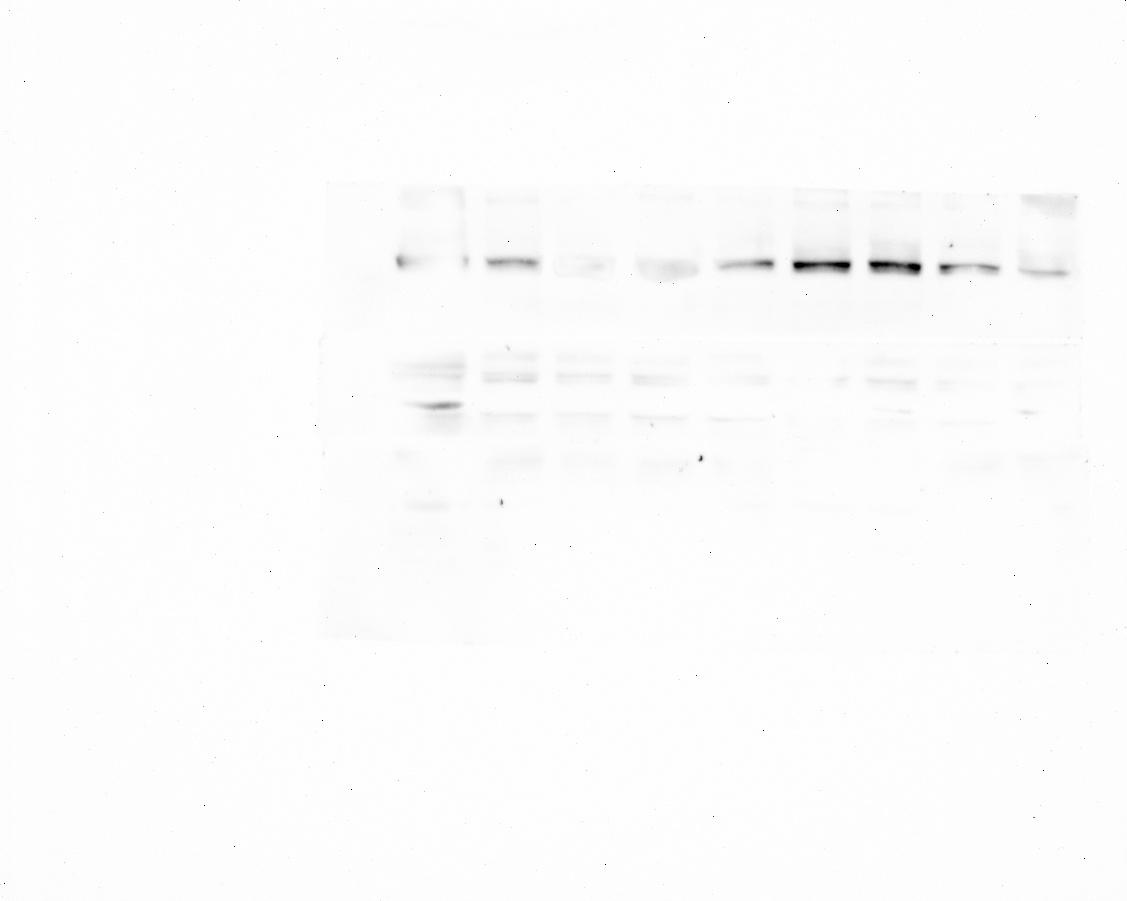

Supplement: Figure 6—source data 3. [file elife-83831-fig6-data3.zip › Figure 6_WB/Fig6d_WB/Fig6d_KO_Serca2a.jpg]

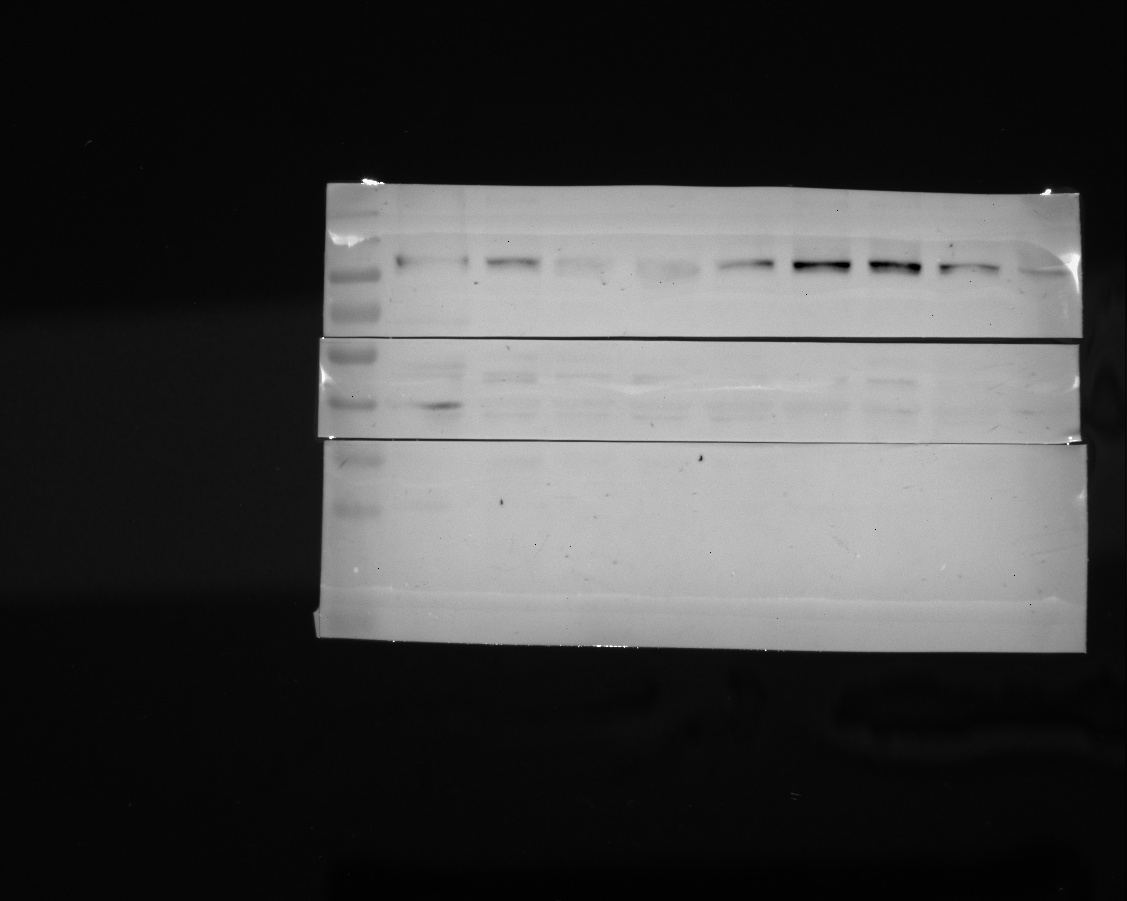

Supplement: Figure 6—source data 3. [file elife-83831-fig6-data3.zip › Figure 6_WB/Fig6d_WB/Fig6d_KO_Serca2a_mbne.jpg]

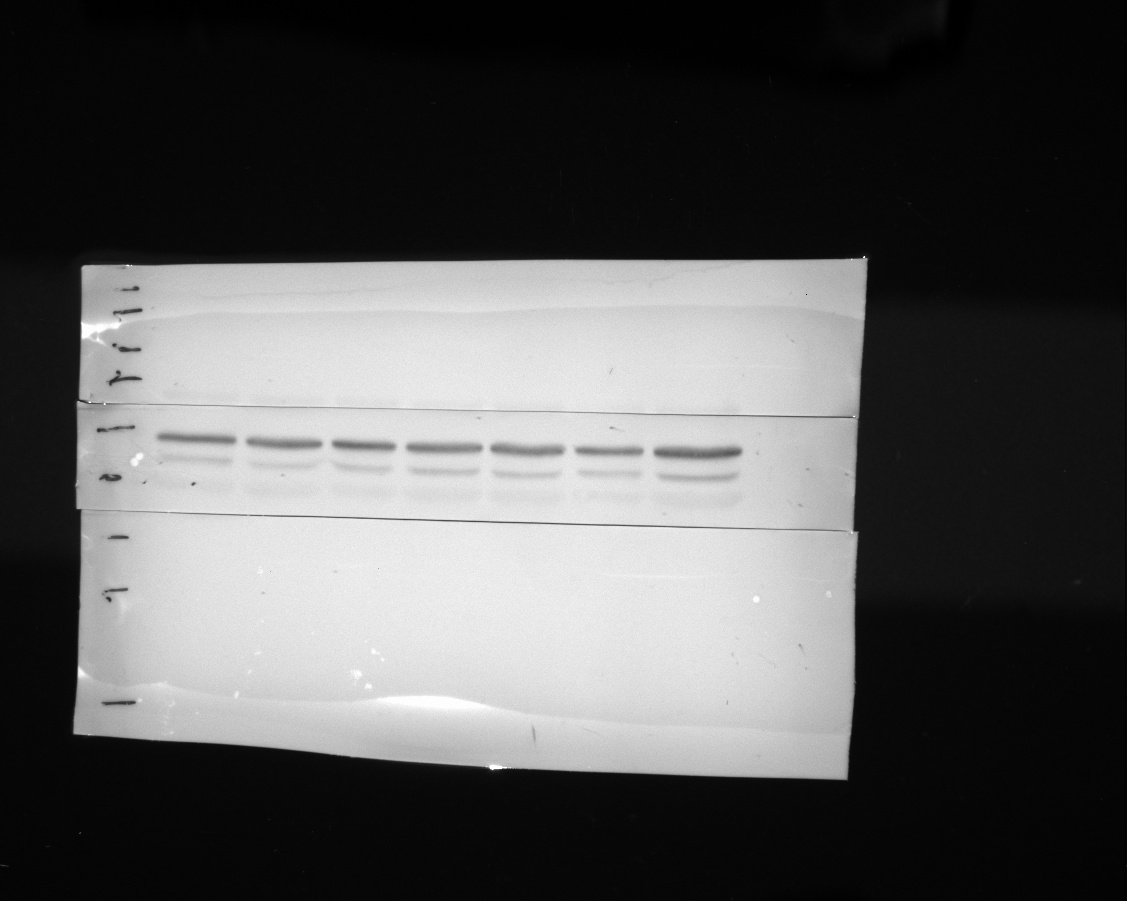

Supplement: Figure 6—source data 3. [file elife-83831-fig6-data3.zip › Figure 6_WB/Fig6d_WB/Fig6d_WT_Calsequestrin.jpg]

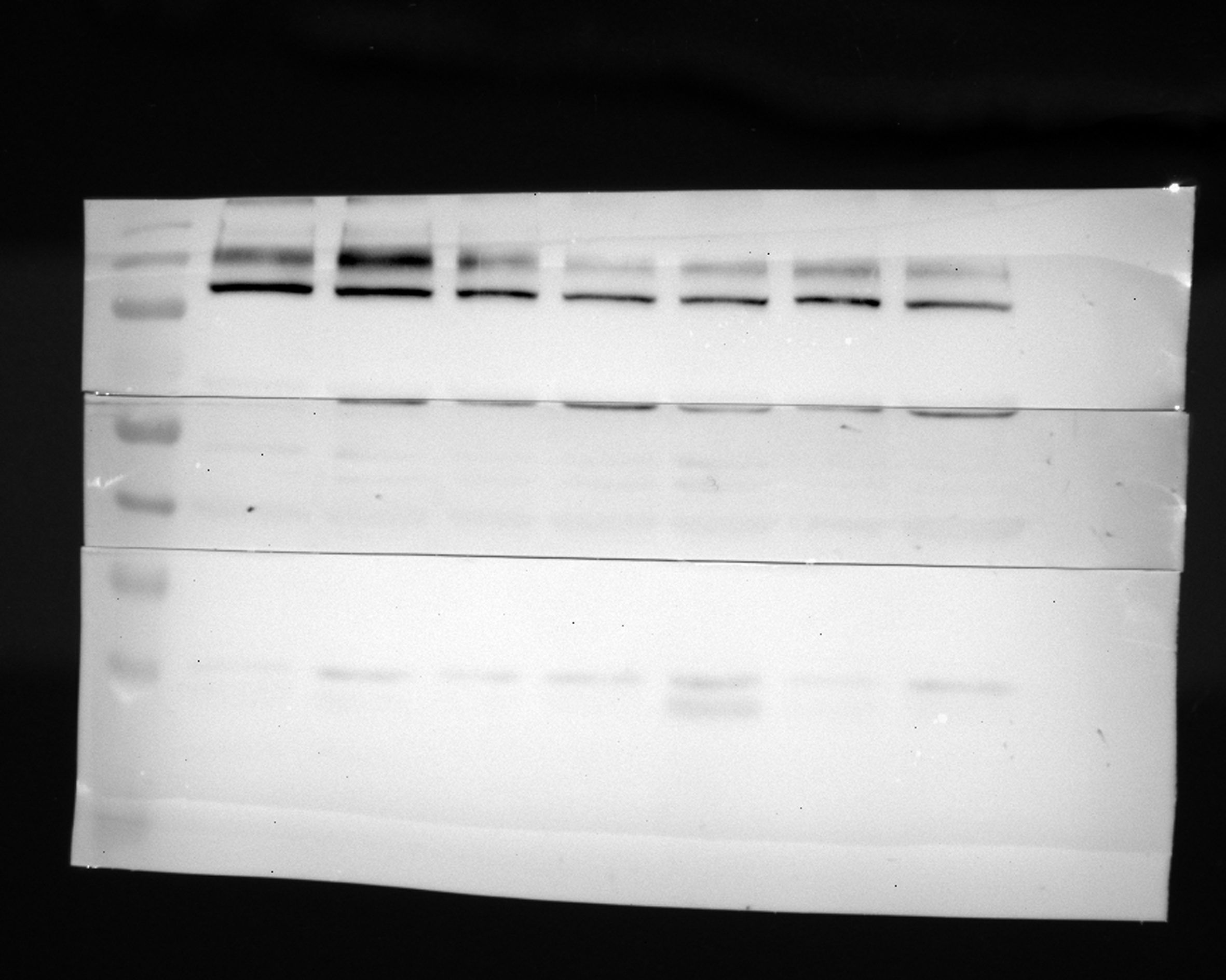

Supplement: Figure 6—source data 3. [file elife-83831-fig6-data3.zip › Figure 6_WB/Fig6d_WB/Fig6d_WT_Serca2.jpg]
